# Zebrafish Blunt-Force TBI Induces Heterogenous Injury Pathologies That Mimic Human TBI and Responds with Sonic Hedgehog-Dependent Cell Proliferation across the Neuroaxis

**DOI:** 10.3390/biomedicines9080861

**Published:** 2021-07-22

**Authors:** James Hentig, Kaylee Cloghessy, Manuela Lahne, Yoo Jin Jung, Rebecca A. Petersen, Ann C. Morris, David R. Hyde

**Affiliations:** 1 Department of Biological Sciences, University of Notre Dame, South Bend, IN 46556, USA; jhentig@nd.edu (J.H.); kcloghes@nd.edu (K.C.); mlahne@nd.edu (M.L.); yjung1@nd.edu (Y.J.J.); 2 Center for Zebrafish Research, University of Notre Dame, South Bend, IN 46556, USA; 3 Center for Stem Cells and Regenerative Medicine, Galvin Life Science Center, University of Notre Dame, South Bend, IN 46556, USA; 4 Department of Biology, University of Kentucky, Lexington, KY 40506, USA; Rebecca.petersen@uky.edu (R.A.P.); ann.morris@uky.edu (A.C.M.)

**Keywords:** traumatic brain injury, blunt-force TBI, regeneration, zebrafish, cerebellum, proliferation, learning, memory

## Abstract

Blunt-force traumatic brain injury (TBI) affects an increasing number of people worldwide as the range of injury severity and heterogeneity of injury pathologies have been recognized. Most current damage models utilize non-regenerative organisms, less common TBI mechanisms (penetrating, chemical, blast), and are limited in scalability of injury severity. We describe a scalable blunt-force TBI model that exhibits a wide range of human clinical pathologies and allows for the study of both injury pathology/progression and mechanisms of regenerative recovery. We modified the Marmarou weight drop model for adult zebrafish, which delivers a scalable injury spanning mild, moderate, and severe phenotypes. Following injury, zebrafish display a wide range of severity-dependent, injury-induced pathologies, including seizures, blood–brain barrier disruption, neuroinflammation, edema, vascular injury, decreased recovery rate, neuronal cell death, sensorimotor difficulties, and cognitive deficits. Injury-induced pathologies rapidly dissipate 4–7 days post-injury as robust cell proliferation is observed across the neuroaxis. In the cerebellum, proliferating *nestin*:GFP-positive cells originated from the cerebellar crest by 60 h post-injury, which then infiltrated into the granule cell layer and differentiated into neurons. Shh pathway genes increased in expression shortly following injury. Injection of the Shh agonist purmorphamine in undamaged fish induced a significant proliferative response, while the proliferative response was inhibited in injured fish treated with cyclopamine, a Shh antagonist. Collectively, these data demonstrate that a scalable blunt-force TBI to adult zebrafish results in many pathologies similar to human TBI, followed by recovery, and neuronal regeneration in a Shh-dependent manner.

## 1. Introduction

Traumatic brain injuries (TBIs) affect every age, ethnicity, and social class in society, with nearly 60 million people affected worldwide annually [1]. However, this number is likely an underestimation, as many people exposed to mild TBIs are often undiagnosed [2,3]. TBIs are categorized by the level of severity (mild, moderate, and severe), which is based on several metrics, including altered state of consciousness, hematoma and edema formation, and structural integrity. TBIs result from either a penetrating or blunt-force trauma. A penetrating trauma results from an object impaling the skull (stab or gunshot wounds) to cause focal brain damage. In contrast, blunt-force trauma, which accounts for over 90% of all TBIs [1,3], arises when the head is struck (motor vehicle accident, fall, sport incident, or combat) to produce a gradient of damage that is relative to the impact force and diffused across the entire brain, often inducing neurodegeneration [4,5,6].

Rodents serve as a powerful TBI model of human injury [7,8,9], with the Marmarou weight drop being a common method to generate a reproducible and scalable TBI [10,11]. This method allows for examining the pathophysiology of blunt-force trauma and the resultant sequelae and changes in cognitive function [10,12,13]. Based on these studies, the TBI-induced phenotypic deficits in rodents are similar to those observed in humans [8]. While mammalian brains possess resident neuronal progenitor cells, they are limited in number and migration potential [14,15,16]. Therefore, they are unable to sufficiently regenerate lost/damaged neurons and restore cognitive function.

Zebrafish, in contrast, possess region-specific populations of resident quiescent stem cells that are induced by injury to regenerate damaged and lost neurons across the nervous system: olfactory system, retina, spinal cord, and brain [17,18,19,20,21,22,23]. The regenerative capacity of the zebrafish brain has been studied following either penetrating TBI, due to either a stab wound or partial excision, by chemical toxins, or ultrasonic or pressure waves [24,25,26,27,28,29]. However, the damage in all these models is primarily focal and/or predominately categorized as severe. Thus, they do not adequately address the heterogeneity of blunt-force TBI severities or phenotypes [30,31]. A zebrafish blunt-force TBI model was recently described to examine changes in gene expression across the brain, but the investigation was limited to a mild injury and failed to address the extent of damage across the brain, the breadth of phenotypic pathologies produced, and the regenerative response [32].

In this study, we describe a scalable blunt-force TBI zebrafish model that recapitulates many of the features of human blunt-force TBI, examines recovery of cognitive function, and describes the extent of neuronal regeneration. We adapted the Marmarou weight drop model [10] to yield a reproducible TBI that is scalable (mild, moderate, and severe). Zebrafish exposed to this modified Marmarou weight drop (MMWD) experienced decreased recovery rate from anesthesia (loss of consciousness), seizures, subdural/intracerebral hematomas, blood–brain barrier disruption, neuroinflammation, cerebral edema, sensorimotor deficits, and learning/memory impairments, which recapitulate key diagnostic pathophysiological features of human TBI. Additionally, we report that the TBI results in widespread cell death across the brain, followed by a proliferative cell response in a severity-dependent manner. Within the cerebellum, injury induced progenitor cells proliferated and migrated over several days, ultimately differentiating into neurons. Upregulation of the Shh ligands, Shha and Shhb, was revealed by qRT-PCR, and we demonstrate that disrupting Shh signaling led to reduced cerebellar injury-induced cell proliferation and reduced production of neurons, while Shh activation induced increased proliferation in undamaged fish. Thus, our scalable zebrafish TBI model may be useful to study the effects of blunt-force trauma, as well as identify key pharmacological and genetic therapeutic targets that regulate injury-induced neuronal regeneration, cognitive recovery, and neuroprotection in an adult vertebrate.

## 2. Materials and Methods

### 2.1. Fish Lines and Maintenance

Adult wild-type AB, *Tg[nestin:GFP]^tud100^* [19], *Tg[gfap:EGFP]^nt11^* [33] transgenic lines, and *albino^b4^* [34] and *roy^a9^;mitfa^w2^* (referred to as *casper*) [35] mutant lines of zebrafish (Danio rerio) were maintained in the Center for Zebrafish Research at the University of Notre Dame Freimann Life Sciences Center. The study used approximately equal numbers of male and female adult zebrafish, 6–12 months old, 3 to 5 cm in length. All experimental protocols in this study were approved by the University of Notre Dame Animal Care and Use Committee protocol #18-03-4558 and adhered to the National Institutes of Health guide for the care and use of Laboratory animals (NIH Publications No. 8023, revised 1978).

### 2.2. TBI Induction via Modified Marmarou Weight Drop

**Figure 1:**
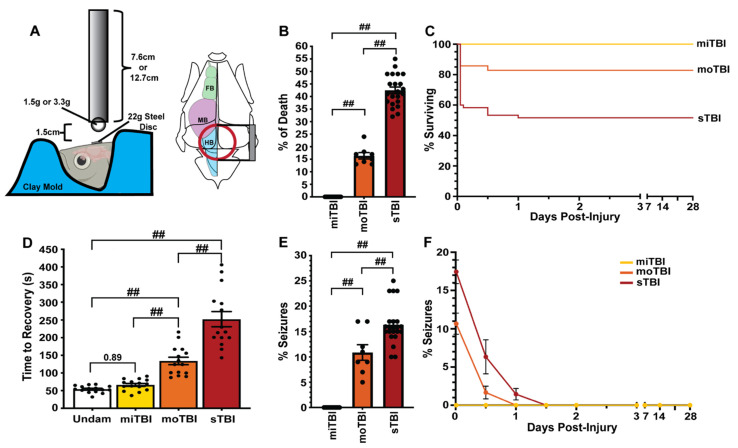
A reproducible and scalable blunt-force TBI in adult zebrafish. (**A**) Diagram depicting the conditions of the modified Marmarou weight drop scaled to the adult zebrafish. On the left, the tube through which the dropping ball falls is shown relative to the head and brain of the zebrafish. On the right, a red circle over the brain diagram shows the relative impact center of the dropping ball (forebrain in green, midbrain in purple, and hindbrain in blue). (**B**) Following damage, the percentage of fish dying significantly increased relative to the damage severity (n ≥ 200 fish). (**C**) Graph of the percentage of fish surviving following TBI out to 28 dpi. All mortality took place within 1 dpi (n = 30). (**D**) The recovery time for the fish to right themselves without exhibiting akinesia, ataxia, and motor incoordination significantly increased with damage severity (n = 15). (**E**) The percentage of fish that experienced intense tonic-clonic seizures significantly increased relative to the damage severity (n ≥ 200 fish). (**F**) The percentage of fish exhibiting post-traumatic seizures observed out to 28 dpi significantly increased relative to the damage severity. No seizures were observed after 1.5 dpi for any of the damage severities (n = 30). Forebrain, FB, hind-brain, HB, midbrain, MB All graph data points are Mean ± SEM. Statistical analyses were performed with either Two-way ANOVA or One-way ANOVA followed by a Tukey post-hoc test. ## *p* < 0.01.

Prior to inducing a blunt-force traumatic brain injury by a modified Marmarou weight drop [10,36], zebrafish were anesthetized in 1:1000 2-phenoxyethanol (2-PE, Sigma-Aldrich, St. Louis, MO, USA) until unresponsive to tail pinch. Anesthetized fish were secured onto a clay mold that stabilized the body and exposed the zebrafish head. To reduce cranial fracture and diffuse the impact, a 22-gauge steel disk was placed onto the skull over the analogous lambda and bregma cranial sutures that are centered over the intersection of the midbrain (mesencephalon and diencephalon; optic tectal lobes) and the hindbrain (rhombencephalon; cerebellum, Figure 1A). Either a 1.5 g or 3.3 g ball bearing weight was dropped down a shaft of either 7.6 or 12.7 cm length, which was placed approximately 1.5 cm above the skull of the fish to produce the desired force (Figure 1A). To induce mild traumatic brain injury (miTBI), a 1.5 g ball was dropped from 9.1 cm (v = 1.34 m/s) producing an energy of 1.33 mJ and an impact force of 1.47 N. A moderate traumatic brain injury (moTBI) was produced by dropping a 1.5 g ball from a height of 14.2 cm (v = 1.91 m/s) resulting in an energy of 2.08 mJ and an impact force of 2.45 N. To induce a severe traumatic brain injury (sTBI), a 3.3 g ball was dropped from 9.1 cm (v = 1.34 m/s) with an energy of 2.94 mJ and an impact force of 3.23 N. Following the TBI, the fish were placed into an aerated tank to recover, monitored for 1 h for seizure activity, and subsequently returned to Freimann Life Sciences Center until further investigation.

### 2.3. Mortality and Early/Latent Seizure

Zebrafish were assessed for survival and post-traumatic seizures within the first hour following blunt-force injury (miTBI, moTBI, sTBI), and at 12 h increments for 30 min at 1, 2, 3, 7, 14, and 28 dpi. Fish were scored for tonic-clonic seizure metrics defined in the zebrafish behavior catalog [37]: ataxia (ZBC 1.9), bending (ZBC 1.16), circling (ZBC 1.32), and corkscrew swimming (ZBC 1.37). At 1 h post-injury (hpi), fish that were unresponsive to tail pinch and displayed no operculum movement for 1 min were considered dead. Mortality and early seizure rates were calculated as an average of at least eight independent experiments (total of 200 fish).

### 2.4. Recovery Rate

Fish were anesthetized and subjected to either no, mild, moderate, or severe blunt-force injury. After 30 s, each fish was returned to a recovery tank and assessed for normal swimming behavior. Recovery rate was calculated as the time interval from entry into the recovery tank until the fish exhibited a complete lack of akinesia (ZBC 1.4), ataxia (ZBC 1.9), and motor incoordination (ZBC 1.99) as defined by Kalueff et al. [37]. The recovery rate of 15 individual fish from three independent trials was averaged. Comparisons were made using a One-way ANOVA followed by a Tukey post-hoc test.

### 2.5. Fluid Content Measurement

The extent of edema was measured using a modified Hoshi protocol [38]. Whole brains (undamaged, miTBI, moTBI, sTBI, n = 3, N = 3, 9 fish total) were extracted from control and injured fish at 1, 3, 5, 7, 14, 28 dpi, weighed wet in weigh boats, and placed in an oven at 60 °C for 12 h. Following drying, each brain was weighed again, and the percent fluid was calculated using the following formula:

(1) $$\%\;\mathrm{Fluid}=\frac{\mathrm{wet}\;\mathrm{weight}-\mathrm{dry}\;\mathrm{weight}}{\mathrm{wet}\;\mathrm{weight}}\;\times \;100\%$$

Comparisons were made using a Two-way ANOVA followed by a Tukey post-hoc test.

### 2.6. Tissue Processing

Undamaged control and blunt-force damaged fish were euthanized by exposure to 1:500 2-PE and brains were removed as described previously [36]. Briefly, fish were pinned dorsal side up on a clay dish, eyes were enucleated, and forceps were inserted behind the lambda suture, removing the right parietal calvarial bone along the sagittal suture. The remaining calvarial bones were removed and the olfactory nerves from the rosette to the olfactory bulbs were bluntly severed. The spinal cord was transected, and the brain was lifted by the caudal end of the spinal cord. Whole brains were extracted and fixed in 9:1 ethanolic formaldehyde (100% ethanol/37% formaldehyde) for 24 h at 4 °C. Brains were rehydrated in a 75%, 50%, and 25% ethanol series for 5 min each at room temperature, transferred into 5% sucrose/PBS for 1.5 h at room temperature, and cryoprotected in 30% sucrose/PBS overnight at 4 °C. Brains were immersed in two parts tissue freezing medium (TFM; VWR International, Radnor, PA, USA) to one part 30% sucrose/PBS for 24 h at 4 °C, and finally 100% TFM for 24 h at 4 °C. Brains were mounted in TFM and stored at −80 °C until cryosectioned. Frozen serial coronal sections of 16 µm thickness were generated for immunohistochemistry/EdU labeling.

### 2.7. Vascular Injury

Undamaged and blunt-force injured (miTBI, moTBI, sTBI) *roy^a9^;mitfa^w2^* (casper) fish were qualitatively assessed at 4 hpi for vascular injury by the presence or absence of subdural pooling. Hematoma formation and resolution was qualitatively assessed in albino^b4^ sTBI fish at 6 and 12 hpi, as well as 1, 1.5, 2, 2.5, and 3 dpi. Brains were prepared as described in the Immunohistochemistry section. Frozen serial coronal sections of 16 μm thickness from rostral tip of the olfactory bulb to caudal aspect of the cerebellum were generated, and intracerebral hematomas were assessed by the presence or absence of blood within serial sections during tissue collection and processing.

### 2.8. Sensorimotor Assay

Undamaged and sTBI fish were assessed at 1, 2, 6, and 12 hpi, as well as 1, 2, 3, 7, 14, and 28 dpi for sensorimotor activity, in regard to swimming orientation and response to a pain stimulus. At appropriate timepoints, individual fish were placed into a 7.5 × 15 cm tank with a water depth of 5 cm and given a 10 min acclimation period. Fish were assessed for swim orientation as defined in the zebrafish behavior catalog [37] (ZBC, 1.83, 1.164, 1.175). Using a 30 g needle, the lateral line was poked, and fish were assessed and scored for: pain response (ZBC 1.104), escape behaviors (ZBC 1.5, 1.52), and avoidance behavior (ZBC 1.12). Each fish was scored for each of the above tests as either responding (score of 1) or not responding (score of 0). The scores for each fish were then summed, such that a fish that exhibited normal swimming orientation and pain response scored a total of 4. A total of 10 fish were analyzed prior to, and following, injury and statistically compared using a repeated measures Kruskal-Wallis test followed by a Dunnett’s multiple comparison post-hoc test.

### 2.9. Locomotion

Undamaged control and blunt-force injured fish (miTIB, moTBI, sTBI, n = 5, N = 3, 15 fish total) were observed at 4 hpi, 1, 2, 3, 4, 7, 14, and 28 dpi. Five fish of the same treatment group were acclimated in a circular observation tank that measured 15 cm in diameter with a water level depth of 10 cm. A digital video recorder, capturing images at a rate of 60 frames per second (fps), was placed above the testing area to monitor the swimming profile of each fish, which was used to quantify the distance that each fish traversed the tank over a 30 s period. Locomotion velocity for each group was calculated (v = swimming distance (m)/30 s) as the average of 15 fish from each treatment group. Comparisons were made using a repeated measures Two-way ANOVA followed by a Dunnett’s multiple comparison post-hoc test.

### 2.10. Shuttle Box Assay Testing Apparatus

A shuttle box assay described by Truong et al. [39] was modified to assess associative learning and memory [40]. The testing apparatus was a modified electrophoresis gel box with a width of 19 cm, length of 30 cm, height of 7.5 cm, and 45-degree ramps on each side leading to an elevated center platform that measured 19 cm × 15 cm, and the water level was set at 5 cm. A standard gel electrophoresis power supply was joined to each tungsten wire to administer a 20 V, 1 mA electrical shock.

### 2.11. Learning

Learning was assessed in the shuttle box assay as previously described [40]. Briefly, undamaged control and blunt-force damaged fish (miTBI, moTBI, sTBI, n = 4, N = 3, 12 fish total) were individually placed into the shuttle box and examined at either 1, 2, 3, 4, 5, 6, 7, 14, or 28 dpi, with no fish being tested twice. After allowing the fish to acclimate in a dark and quiet room for 15 min, the fish was exposed to a red light visual stimulus that was placed against the plexiglass tank that contained the fish. The visual stimulus was applied alone for 15 s. If the fish swam to the other stall in response to the visual stimulus, the red light was turned off once it passed the half-way mark of the tank. However, if the fish failed to swim to the other stall, the red light was kept on and a pulsating electric current (20 V, 1 mA) was simultaneously applied until either the fish swam to the other stall or for 15 s (10 electrical shocks/15 s), whichever came first. The presentation of a light stimulus/electrical shock was repeated with 30 s rest intervals. Learning was defined as the fish successfully swimming across the tank within 15 s of light presentation on 5 consecutive trials. The number of trials that each fish required to learn were determined for each treatment group and averaged for each experiment. A Two-way ANOVA and Tukey’s post-hoc test was performed to statistically compare the undamaged control fish and the different damage groups.

### 2.12. Immediate and Delayed Recall

Recall was assessed in the shuttle box assay as previously described [40]. Briefly, undamaged fish were individually placed into the shuttle box testing apparatus and tested as described under Learning. Fish were trained by 25 repetitions of exposure to red light and if they failed to swim to the other tank in response to the red light stimulus they were electrically shocked. Following this training period, fish were given 15 min to rest before they were tested 25 times by exposing the fish only to the red light stimulus (testing period 1). Each test involved applying the visual stimulus alone for 15 s. If the fish swam to the other stall within the initial 15 s, the red light was turned off after passing the half-way point of the tank and this was scored as a successful trial. However, if the fish failed to swim to the other stall, the pulsating electric current was simultaneously applied either until the fish swam to the other stall or for 15 s (10 shocks/15 s). The number of successful tests, defined as the fish crossing the tank without the electric shock, were counted to generate the initial testing baseline. Once all experimental fish were tested, they were randomly selected for either the undamaged control group or were administered a blunt-force injury (miTBI, moTBI, or sTBI). To assess immediate recall, the control group and TBI groups were retested either 4 h after the initial testing period or 4 hpi, respectively. All groups were subjected to a second testing period consisting of 25 iterations and scored for the number of successful tests. To test delayed recall, the undamaged fish were returned to Freimann Life Sciences Center after the initial testing period for four days. Fish were then randomly selected for either the undamaged control group or were administered a blunt-force injury (miTBI, moTBI, sTBI) and allowed to recover for 4 h. The fish were then subjected to a second testing period of 25 iterations and scored for the number of successful trials as described above. For both immediate and delayed recall, the percent difference in the number of successful secondary trials relative to the number of initial successful trials was calculated and averaged for each group (n = 3, N = 3, 9 fish total). Undamaged control and the different damaged groups were statistically assessed using a Two-way ANOVA and Tukey’s post-hoc test.

### 2.13. Startle Response Habituation

Undamaged and sTBI fish (n = 3, N = 3, 9 fish total) were assayed for the startle response at 1, 4, and 7 dpi using a modified protocol from Chanin et al. [41]. Individual fish were placed in a testing tank (31 cm long × 19 cm wide × 15 cm high with water level depth of 10 cm) and allowed to acclimate for 15 min. A digital video recorder, capturing images at a rate of 60 fps, was placed above the testing area to monitor the swimming profile of each fish. A 100 g weight was dropped from 15 cm at the water level every 60 s for 10 iterations. The initial velocity following startle, velocity for each second, the time to recovery from the startle, and the total distance that each fish traversed the tank over 60 s following the startle was quantified. A One-way ANOVA followed by a Tukey’s post-hoc test was used to statistically compare total distance swam by undamaged controls and sTBI fish.

### 2.14. Terminal Deoxynucleotidyl Transferase dUTP Nick-End Labeling (TUNEL) Assay

Undamaged control and blunt-force damaged fish at 16 hpi were anesthetized in 1:1000 2-PE until unresponsive to tail pinch. Fish were placed with the ventral side facing up, secured, and transcardially perfused with PBS (1 mL/1 min) for 5 min. Brains were removed from the euthanized fish and fixed in 9:1 ethanolic formaldehyde (100% ethanol/37% formaldehyde) for 24 h at 4 °C. For fluorescently labeled TUNEL: Brains were cryoprotected as described in the ‘Tissue processing’ section, and frozen serial coronal sections of 16 μm thickness of the entire brain were collected. Cell death was detected using ApopTag^®^ Red In Situ Apoptosis Detection Kit (EMD Millipore, Burlington, MA, USA) following the manufacturer’s instructions with the following deviations: Prior to labeling, cryosections were dried at 55 °C for 20 min then post-fixed for 30 min in 2% PFA, rehydrated in PBS, and permeabilized in PBS containing 0.5% Triton X-100 for 5 min at room temperature. The sections were then exposed to acetic acid:EtOH at −20 °C for 5 min, followed by an incubation in Proteinase K 10 mg/mL (1:200 dilution in PBS) for 20 min at room temperature, washed briefly in PBS for 5 min, and then incubated with equilibration buffer for 3 min at room temperature. Apoptotic cells were incubated with the manufacturer’s TdT label mix at 37 °C for 1.5 h. To stop the enzymatic reaction, slides were incubated in 1× stop buffer for 10 min and then washed in PBS before TUNEL-positive cells were detected by a Dig-based system. Slides were incubated in a 1:1 ratio of anti-dig/blocker for 30 min, washed in PBS twice for 5 min each before slides were incubated in DAPI (1:1000) for 20 min. Slides were then repermeabilized in PBS-Tween 20 for 10 min, blocked in PBS containing 2% normal goat serum, 0.4% Triton X-100, and 1% DMSO, and incubated in primary rabbit anti-HuCD (1:100, Abcam, Cambridge, MA, USA) antibody diluted in blocking buffer in a humidity chamber at room temperature overnight. Slides were then rinsed three times in PBS-Tween-20 for 10 min each, and subsequently incubated in fluorescent-labeled secondary goat anti-rabbit antibody (diluted 1:500 in blocker) and DAPI (1:1000) in a humidity chamber at room temperature for 1.5 h. Slides were then briefly rinsed in PBS for 5 min before being mounted in ProLong Gold Antifade (Life Technologies, Carlsbad, CA, USA).

For DAB labeled TUNEL: Undamaged control and blunt-force damaged fish at 16 hpi were anesthetized in 1:1000 2-PE until unresponsive to tail pinch. Brains were perfused and fixed as described above for fluorescently labeled TUNEL. Whole heads were then decalcified in 100% filtered RDO Rapid Decalcifier (Electron Microscopy Sciences, Hatfield, PA, USA) for 1 h at room temperature. Brains were then dehydrated in a 70%, 80%, 90%, 95% ethanol series for 1 h each, followed by three 1 h exposures to 100% ethanol and xylene each. Subsequently, whole heads were submersed twice for 1 h each under vacuum in 60 °C paraffin. Whole heads were embedded in paraffin and 10 μm thick serial sections were prepared. Apoptotic cell death was visualized using NeuroTACS II In Situ Apop Detection Kit (Trevigen, Gaithersburg, MD, USA). Slides were deparaffinized in three xylene washes and rehydrated by incubating slides twice each in 100%, 95%, 70% ethanol for 5 min. Slides were washed for 10 min at room temperature in PBS and coated with the manufacturer’s NeuroPore^®^ overnight. Slides were rinsed in PBS for 10 min and quenched in 9:1 methanol: 30% hydrogen peroxide for 5 min. Slides were washed in PBS for 10 min, coated with manufacturer’s TdT label buffer for 5 min, followed by manufacturer’s label reaction mix for 1 h at 37 °C. To stop the enzymatic reaction, 1× TdT stop buffer was applied for 5 min and slides were then washed in PBS for 5 min. Slides were covered with Strep-HRP Solution at room temperature for 20 min, washed in PBS for 5 min, and then submerged in DAB solution for 4 min. Slides were exposed to 95% ethanol once, 100% ethanol twice, and xylene twice for 3 min each to dehydrate the tissue. Subsequently, slides were coverslipped with DPX (Sigma-Aldrich, St. Louis, MO, USA) to preserve DAB staining.

### 2.15. EdU Labeling

Fish were anesthetized in 1:1000 2-PE and intraperitoneally injected (IP) with ~40 µL of 10 mM 5-ethynyl-2′-deoxyuridine (EdU, Invitrogen, Carlsbad, CA, USA) using a 30-gauge needle. For cell migration experiments, undamaged control and blunt-force injured fish were injected with EdU at 48 hpi and collected at either 51, 60, 72, 84, or 96 hpi (n = 4, N = 3, 12 fish total). For recovery experiments, undamaged control and blunt-force injured fish were injected with EdU at 48 hpi and 60 hpi and collected at either 7 or 30 dpi (n = 3–5, N = 3, 10–15 fish total). For all other experiments utilizing EdU, undamaged control and blunt-force injured fish were injected at 48 hpi and collected at 60 hpi (N = 1–4 in triplicate, N total = 4–10). Tissue was collected, fixed, and sectioned as described in the ‘Tissue processing’ section. EdU detection was performed using the Click-iT^®^ EdU Alexa Fluor^®^ 594 Imaging Kit (Invitrogen, Carlsbad, CA, USA) as previously described [42,43], which was performed prior to immunohistochemistry.

### 2.16. Immunohistochemistry

Brain cryosections that were prepared as described in the ‘Tissue processing’ section (16 μm) were dried at 55 °C for 20 min before being rehydrated and permeabilized in PBS-Tween 20 (0.05%, Sigma-Aldrich, St. Louis, MO, USA) for 10 min. Sections were blocked in PBS containing 2% normal goat serum, 0.4% Triton X-100, and 1% DMSO for 1 h in a humidity chamber at room temperature. Slides were incubated in primary antibody diluted in blocking buffer in a humidity chamber at room temperature overnight. Primary antibodies (and their dilutions) included mouse anti-GFP monoclonal antibody (1:500, Sigma-Aldrich, St. Louis, MO, USA), chicken anti-GFP polyclonal antibody (1:500 for brain, 1:1000 for retina, Abcam, Cambridge, MA, USA), rabbit anti-PCNA polyclonal antibody (1:2000, Abcam, Cambridge, MA, USA), and rabbit anti-HuC/D polyclonal antibody (1:100, Abcam, Cambridge, MA, USA). Slides were then rinsed three times in PBS-Tween-20 for 10 min each, and subsequently incubated in fluorescent-labeled secondary antibody (diluted 1:500 in PBS-Tween-20) and DAPI (1:1000) in a humidity chamber at room temperature for 1.5 h. The secondary antibodies used in this study included either goat anti-mouse IgG or goat anti-rabbit IgG conjugated to Alexa Fluor 488, 594, or 647 and goat anti-chicken IgG conjugated to Alexa Fluor 488 (Jackson Immunoresearch Laboratories, West Grove, PA, USA). Sections were rinsed three times in PBS-Tween 20 for 10 min each and once in PBS for 5 min before being cover-slipped with ProLong Gold Antifade (Life Technologies, Carlsbad, CA, USA).

### 2.17. Image Acquisition

Confocal z-stacked images of 16 µm sections were taken every other section throughout the cerebellum. Z-stacks of 10 µm thickness were acquired in 0.5 µm steps, imaged at 1024 × 1024 on a Nikon A1R microscope using either a 20× (NA 0.75) or 40× (NA 1.3) oil-immersion objective. Channel crosstalk was minimized by acquiring images using the sequential channel series function (NIS-Elements 4.13.01, 5.20.02 software). Images across the entire brain were taken every 5th section and acquired using a 20× oil-immersion objective (NA 0.75) and employing the large-image acquisition function (15% overlap, NIS Elements). Brightfield images of DAB-TUNEL labeled sections were acquired using a Nikon 90i microscope equipped with a 20× objective (NA 0.75) lens and a color camera (NIS-Elements AR 3.2 software). Lightsheet images were collected on a Z.1 Dual Illumination Lightsheet using a 5× objective with a refractive index of 1.45. Images were acquired in Multiview as 2 × 3 tiles. The image tiles were stitched together and rendered in the arivis Vision4D software to form the final images.

### 2.18. Tissue Clearing/EdU Labeling

We modified the brain clearing protocol described by Lindsey et al. [44]. EdU was intraperitoneally injected into undamaged control and sTBI fish (as described above) and transcardially perfused at 60 hpi with 2% paraformaldehyde (PFA,1 mL/min) for 3 min. Brains were collected and fixed in 2% PFA at 4 °C overnight. Brains were washed four times in 0.3% Triton X-100/PBS for 30 min each on a shaker and then permeabilized in 1% Triton X-100/5% DMSO/PBS for 24 h on a shaker at 4 °C, and then washed four times for 30 min in 0.3% Triton X-100/PBS. EdU was labeled as previously described by Lindsey (3 days of labeling) [44]. Brains were washed four times in cold PBS, for 30 min each, to remove unbound fluorescently conjugated azide. Subsequently, brains were embedded in 1% low melting point agarose, dehydrated in 100% MeOH four times for 4 h each, and cleared during four washes in 2:1 benzyl benzoate and benzyl alcohol (Sigma-Aldrich, St. Louis, MO, USA) that lasted 4 h each.

### 2.19. Optical Density

Images of EdU-labeled undamaged and sTBI cleared brains at 60 hpi were taken at 40× using a Leica M205 FA epifluorescent microscope (Leica Application Suite 2.2.0 build 4765 software). Using ImageJ software (National Institutes of Health, Bethesda, MD, USA), images were converted to 8-bit gray scale and the gray area intensities were individually determined for each of the major brain regions (forebrain, midbrain, hindbrain). The background gray area intensity was measured within a region of interest (ROI) of each brain region that was placed at the outside edge, which did not contain a discernable focal point of fluorescence within its boundary of the background [22]. The optical density was calculated for each fish using the following formula:

(2) $$\mathrm{OD}=\log\;\frac{\mathrm{Background}\;\mathrm{intensity}}{\mathrm{avg}.\;\mathrm{intensity}\;\mathrm{of}\;\mathrm{ROI}}$$

Comparisons across groups were made using a Two-way ANOVA followed by a Tukey’s post-hoc test.

### 2.20. Evans Blue Assay for Blood–Brain Barrier Disruption

Using modified Radu et al. and Eliceiri et al. protocols [45,46], undamaged and TBI injured fish (miTBI, moTBI, sTBI, n = 3, N = 3, 9 fish total) were intracardiacly injected immediately following sham/TBI, or at desired time point, with 50 µL of 1% Evans blue dye (Sigma-Aldrich, St. Louis, MO, USA) in PBS. Fish were allowed to survive for 2 h post-injection. Fish were then euthanized in 1:500 2-PE, brains were collected, weighed, and washed in PBS for 3 min to remove excess and superficial dye accumulated during dissection. For quantitative assays, 3 brains from a treatment group were pooled, placed in 300 µL of 100% formamide and incubated at 55 °C for 24 h. Brains were then centrifuged at 10× *g* for 10 min, the supernatant collected, 250 µL from each group was analyzed on a SpectraMax M5 plate reader (Molecular Devices, San Jose, CA, USA), and RFU value was normalized to mg of brain tissue. Comparisons were made using a One-way ANOVA followed by either a Tukey’s or Dunnett’s multiple comparison post-hoc test. For qualitative sections, brains were fixed in 4% PFA overnight. Brains were then embedded in 5% low melt agarose and a single coronal cut was made with a razor blade, and en face coronal images were taken.

### 2.21. Sonic Hedgehog Modulation

Sonic hedgehog signaling was modulated with either an agonist (purmorphamine, Sigma-Aldrich, St. Louis, MO, USA) or an antagonist (cyclopamine, Toronto Research Chemicals, Toronto, ON, Canada). Purmorphamine: Undamaged fish were IP-injected with 40 µL of 10 µM purmorphamine using a 30-gauge injection needle attached to a 1 mL syringe every 12 h for 48 h (0, 12, 24, 36, and 48 h). To assess cell proliferation in undamaged control and purmorphamine treated brains, EdU was coinjected (as described above) at the 48 h timepoint. Brains were collected 12 h later (60 h after the first purmorphamine injection) and prepared for EdU detection and immunohistochemistry as described above. Cyclopamine: Fish exposed to sTBI were IP-injected with 40 µL of 2 mM cyclopamine at 4, 12, 24, 36 hpi using a 30-gauge injection needle attached to a 1 mL syringe and coinjected with EdU at 48 hpi. Brains were collected at 60 hpi and prepared for EdU detection and immunohistochemistry as described above.

### 2.22. Quantitative Real-Time PCR (qRT-PCR)

Total RNA was isolated and purified from approximately the top 1/3 apical portion of cerebellums from five adult undamaged and sTBI fish. For neuroinflammation investigations, sTBI fish were collected at 12 hpi, 1, 2, 3, 7, 14, and 28 dpi using Trizol extraction and converted to cDNA from 1 ug of RNA using qScript cDNA SuperMix (Quantabio, Beverly, MA, USA) as described by Campbell et al. [47], and TaqMan probes were used according to manufacturer’s instructions, with 10 ng of cDNA for amplification. TaqMan probes (Thermo Fisher, Waltham, MA, USA) for *il1β* (Dr03114367_g1), *tnf*α (Dr03126850_m1), and *il10* (Dr03103209_m1) were used to examine neuroinflammation. For sonic hedgehog investigations, sTBI fish were at 6, 12, 24, 36, 48, and 60 hpi, and TaqMan probes (Thermo Fisher, Waltham, MA, USA) for *shha* (Dr03432632_m1), *shhb* (Dr03112045_m1), *smo* (Dr03131349_m1), and *gli1* (Dr03093665_m1) were used. For quantitative real-time PCR (qRT-PCR) the data was normalized to 18s rRNA (Hs03003631_g1) in each well. Data was acquired using the ABI StepOnePlus Real-Time PCR System (Applied Biosystems, Foster City, CA, USA). Cycling conditions were as follows: 2 min at 50 °C, 10 min at 95 °C, 40 cycles of 15 s at 95 °C, and 1 min at 60 °C with data collection occurring after each extension cycle. The ΔΔCT values were calculated and used to determine the log_2_-fold changes [33] of *il1β*, *tnf*α, *il10*, *shha*, *shhb*, *smo*, and *gli1*. Expression levels were examined in biological triplicate and technical replicates.

### 2.23. Statistical Analysis

All data within this study, with the exception of sTBI Blood-Brain Barrier disruption time course (n = 3 pooled brains, N = 2), whole brain fluorescent TUNEL (n = 1), whole brain proliferation (n = 2, N = 2, total of 4 fish), was obtained from at least three independent trials (N = 3) of at least 3 fish per independent trial (n = 3, total of 9 fish). The data are expressed as mean ± SE or as mean ± SD, each indicated within the figure legend, which was derived by averaging the data from the brains of individual fish from all combined trials. Data sets were analyzed in Prism 8 (GraphPad, San Diego, CA, USA) with a Student’s *t*-test for single pairwise comparisons with control or One-way or Two-way ANOVA followed by either Tukey’s, Bonferroni’s, Dunnett’s, or Sidik’s post-hoc test for multiple comparisons. The statistical test used and significance indicator of # for *p* < 0.05 or ## for *p* < 0.01 are stated in each figure legend. In instances where comparisons were not statistically significant, the actual *p*-value was given in the figure.

## 3. Results

### 3.1. Modified Marmarou Weight Drop Results in a Reproducible and Scalable TBI

Blunt-force injuries, the most common form of TBI, range in severity and result in a heterogeneous set of injury-induced pathologies. While a blunt-force zebrafish TBI model has rarely been examined [32] it offers the unique opportunity to also examine the regenerative response. To develop a scalable blunt-force TBI model, we modified the commonly used Marmarou weight drop (Figure 1A, created with BioRender.com) [10,36], with the impact zone centered at the intersection of the midbrain (mesencephalon and diencephalon; optic tectal lobes) and hindbrain (rhombencephalon; cerebellum, Figure 1A).

To validate our model, we examined key pathophysiological features often used to categorize TBI, such as mortality, loss of consciousness, seizure, vascular injury, blood–brain barrier (BBB) disruption, edema, sensorimotor deficits, and neuroinflammation [3,48,49]. We determined the percentage of mortality for each level of damage. A mild traumatic brain injury (miTBI) never resulted in death (n = 225, Figure 1B). However, mortality significantly increased (16.37% ± 1.28%, *p* < 0.01, n = 143, Figure 1B) when the severity was increased to moderate TBI (moTBI) and was further significantly elevated (42.45% ± 1.33%, *p* < 0.01, n = 938, Figure 1B) following a severe TBI (sTBI). While we continued to monitor survival for 28 days post-injury (dpi), all mortality was observed within 1 dpi (Figure 1C). Thus, our model resulted in injuries with reproducible high, medium, and low survival rates, correlative to the prognostic outcomes in humans suffering from all three severity levels [1,49].

We next examined loss of consciousness, a commonly used diagnostic measure to rapidly categorize human TBI [50], by quantifying the amount of time required before returning to normal swimming behavior immediately following injury. Undamaged controls rapidly recovered from anesthesia in the recovery tank (Undam: 52 s ± 2.57 s) and the recovery rate following miTBI was not significantly different (61 s ± 4.1 s, *p* = 0.89, n = 15, Figure 1D). However, moTBI fish took significantly more time (133.93 s ± 10.56 s, *p* < 0.01, n = 15, Figure 1D) relative to undamaged controls before they regained consciousness and returned to normal swimming behavior. The sTBI fish took significantly longer than either undamaged control, miTBI, or moTBI fish, as they often remained motionless at the bottom of the tank for several minutes (252.2 s ± 21.19 s, *p* < 0.01, n = 15, Figure 1D). Thus, our model displayed increased times of lack of consciousness that were consistent with the level of TBI severity.

We also determined the percentage of zebrafish that displayed intense tonic-clonic seizure-like behaviors (akinesia, ataxia, bending, circling, and/or corkscrew swimming). This seizure-like behavior was never observed following a miTBI (n = 225 Figure 1E). However, the percentage of fish exhibiting tonic-clonic seizures significantly increased following moTBI (10.87% ± 1.54%, *p* < 0.01, n = 143 Figure 1E) relative to miTBI fish, and significantly increased further in sTBI fish (16.63% ± 0.84%, *p* < 0.01, n = 938, Figure 1E). Additionally, injured fish were observed for post-traumatic seizure activity from time of injury out to 28 dpi (Figure 1F). Following moTBI and sTBI, seizure activity was observed for 1 (moTBI) to 1.5 dpi (sTBI), after which all seizure behavior ceased and was not observed again through 28 dpi (Figure 1F). The increase in number of seizures relative to the injury severity observed in our model is in agreement with human blunt-force TBI populations [51,52].

### 3.2. Blunt-Force TBI Induces Severity-Dependent Vascular Injury with Blood–Brain Barrier Disruption, Neuroinflammation, and Edema

**Figure 2:**
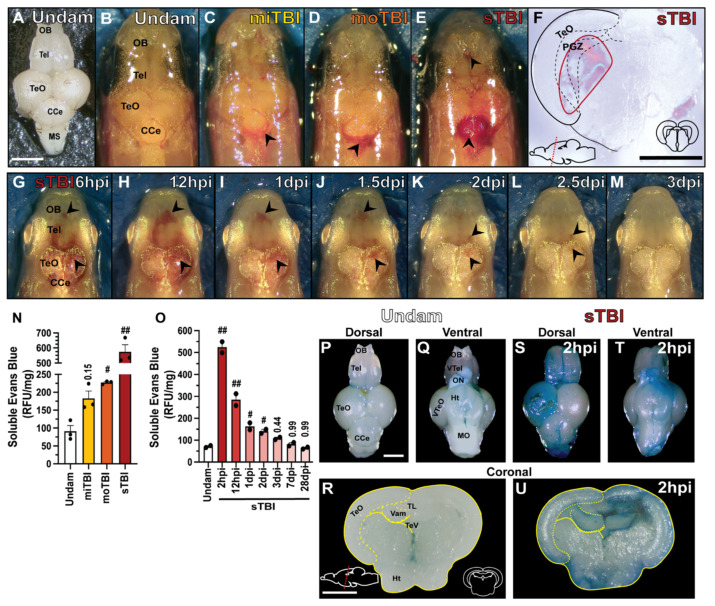
MMWD produces graded hematomas and blood–brain barrier disruption. (**A**) Isolated, undamaged whole brain with the major lobes labeled. Dorsal views of *roy^a9^;mitfa^w2^* (*casper*) undamaged (**B**) and TBI fish displaying vascular injury 4 hpi (**C**–**E**). Compared to undamaged controls, vascular injury resulted in hemorrhaging (arrowheads) in all severity levels that increased in a severity-dependent manner. (**F**) Coronal section of sTBI brain with intracerebral hematoma (red boundary). (**G**–**M**) Repeated dorsal view of an individual sTBI *albino^b4^* fish across time, in which hemorrhaging (arrowheads) qualitatively peaked at 12 hpi, and gradually resolved by 3 dpi. (**N**) Following injury, a significant increase in solubilized Evans Blue dye represented disruption of the BBB in a severity-dependent manner. (**O**) Following sTBI, a statistically significant increase in solubilized Evans Blue dye occurred by 2 hpi and then gradually decreased until it reached control levels at 3 dpi (3 pooled brains/group, n = 2–3 groups). (**P**–**U**) Dorsal, ventral, and coronal views of isolated undamaged (**P**,**Q**,**R**) and sTBI brains at 2 hpi (**S**,**T**,**U**) from fish injected with Evans Blue dye as a qualitative measure of BBB integrity. Solid lines in (**F**,**R**,**U**) denote tissue boundaries, while dotted lines denote internal anatomical boundaries. Corpus cerebelli, CCe, hypothalamus, Ht, medial valvula cerebelli, Vam, medulla oblongata, MO, medulla spinalis, MS, olfactory bulb, OB, optic tectum, TeO, periventricular grey zone, PGZ, tectal ventrical, TeV, telencephalon, Tel, torus longitudinalis, TL, ventral optic tectum, VTeO, ventral telencephalon, VTel. Scale bars, (**A**–**E**,**G**–**M**,**P**–**T**) = 500 µm, (**F**,**R**–**U**) = 250 µm. Mean ± SEM is depicted in (**N**,**O**). Statistical analyses were performed with a One-way ANOVA followed by a Tukey post-hoc test. ## *p* < 0.01.

In human TBI, vascular injury, blood–brain barrier (BBB) disruption, edema, and neuroinflammation are critical metrics [53,54]. At 4 hpi, miTBI fish displayed minor bleeding (Figure 2C, arrowheads) that was not observed in undamaged controls (Figure 2A,B). As injury severity increased, we observed apparent pooling of blood following both moTBI and sTBI (Figure 2D,E), with intracerebral hematomas and blood-filled ventricles found in sTBI fish (Figure 2F). In sTBI fish, hematoma formation continued to expand between 6 and 12 hpi (Figure 2G,H), with hematomas visually resolving between 1–3 dpi (Figure 2I–M). We further assessed vascular injury in terms of BBB disruption using Evans blue dye [46] that was intracardiacly injected and permeated into the brain for 2 hpi. Undamaged and miTBI fish had low amounts of Evans blue diffuse across the BBB (91.18 RFU/mg ± 15.75 and 182.93 RFU/mg ± 20.68, *p* = 0.15, respectively, Figure 2N,P,Q). In contrast moTBI and sTBI had significantly more Evans blue dye diffuse across the BBB (moTBI: 227.16 RFU/mg ± 4.38, *p* < 0.05, sTBI: 574.26 RFU/mg ± 47.49, *p* < 0.01 Figure 2N,S,T). The significant increase of Evans blue dye in the sTBI fish was apparent in coronal brain sections (Figure 2U) relative to undamaged controls (Figure 2R). We also measured BBB disruption using Evans blue dye in sTBI fish from 2 hpi through 28 dpi. BBB disruption sharply peaked by 2hpi and remained significantly disrupted through 2 dpi (2 hpi: 524.01 RFU/mg, *p* < 0.01, 12 hpi: 285.06 RFU/mg, *p* < 0.01, 1 dpi: 163.49 RFU/mg, *p* < 0.05, 2 dpi: 140.72 RFU/mg, *p* < 0.05, Figure 2O). Extracted dye levels returned to near undamaged levels by 3 dpi and remained there through 28 dpi (undam: 70.73 RFU/mg, 3 dpi: 108.02 RFU/mg, *p* = 0.44, 7 dpi: 81.79 RFU/mg, *p* = 0.99, 28 dpi: 63.94 RFU/mg, *p* = 0.99, Figure 2O). The observed hematoma growth and BBB disruption led us to investigate edema and neuroinflammation.

**Figure 3:**
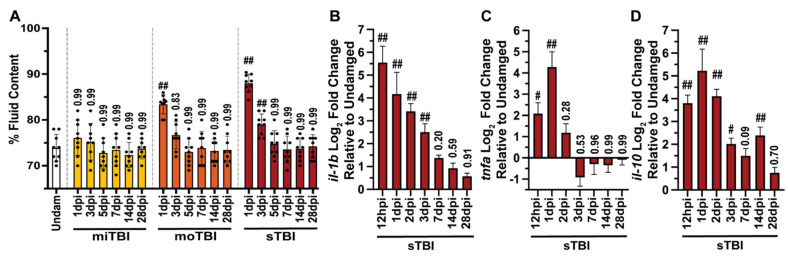
TBI inflicted zebrafish display edema and neuroinflammation. (**A**) Graph of level of edema, measured as the percentage of fluid in the brain, at all severities from 1–28 dpi, in which edema increased in a severity-dependent fashion, and remained elevated until 3 dpi and 5 dpi for moTBI and sTBI, respectively (n = 9). (**B**,**C**) Expression of pro-inflammatory cytokine genes *il-1b* (**B**) and *tnfa* (**C**) were significantly elevated in sTBI for 3 or 1 dpi, respectively, following injury before returning to near undamaged levels (5 pooled cerebellums/group, n = 3 groups). (**D**) Anti-inflammatory cytokine *il-10* gene expression was also elevated and remained highly upregulated for 14 dpi (5 pooled cerebellums/group, n = 3 groups). Mean ± SEM is depicted in all graphs. Statistical analyses were performed with a One-way ANOVA followed by a Tukey’s or Dunnett’s multiple comparison post-hoc test. # *p <* 0.05, ## *p* < 0.01.

Edema formation, which was measured as the percentage of fluid in the brain, was not significantly different between undamaged controls and miTBI fish between 1 through 28 dpi (Undam: 76.36% ± 0.98%, miTBI 1 dpi: 76.11% ± 0.93%, *p* = 0.99, n = 9, Figure 3A). However, as injury severity increased, so did the percentage of fluid content of damaged brains, indicative of edemas. Relative to undamaged controls, both the moTBI (83.42% ± 0.83%, *p* < 0.01, n = 9) and sTBI (88.02% ± 0.64%, *p* < 0.01, n = 9, Figure 3A) brains contained significantly more fluid at 1 dpi. Increased edema remained significantly elevated in sTBI fish at 3 dpi (79.02% ± 0.76%, *p* < 0.01), while moTBI returned near undamaged levels (76.66% ± 0.99%, *p* = 0.83, Figure 3A). By 5 dpi, edema within sTBI brains returned to near undamaged levels (74.77 ± 0.95%, *p* = 0.99) and significantly elevated edema was not again observed in any severity out through 28 dpi (Figure 3A).

We also assessed neuroinflammation by examining representative pro-inflammatory (*il1β, tnf*α) and anti-inflammatory (*il10*) cytokine expression using qRT-PCR of RNA collected from undamaged and sTBI isolated cerebellums across multiple timepoints following blunt force trauma (12 hpi, 1, 2, 3, 7, 14, and 28 dpi). The cytokines *il1β*, *tnf*α, and *il10* have been implicated as critical biomarkers in human TBI [55,56,57] and as playing a role in zebrafish tissue regeneration [42,58]. Relative to undamaged control cerebellums, *il1β* expression peaked at 12 hpi and remained highly upregulated through 3 dpi (Figure 3B), while *tnf*α expression peaked at 1 dpi, before rapidly decreasing and returning near undamaged levels (Figure 3C). The anti-inflammatory cytokine *il10* also began increasing by 12 hpi and remained elevated through 14 dpi before returning to near undamaged levels by 28 dpi (Figure 3D). Collectively, these data demonstrate our model recapitulates several injury-related pathologies consistent with key human diagnostic TBI measures and provides the sensitivity to reproducibly distinguish between mild, moderate, and severe TBI.

### 3.3. Blunt-Force TBI Results in Severity-Dependent Cell Death Spreading from the Impact Zone

**Figure 4:**
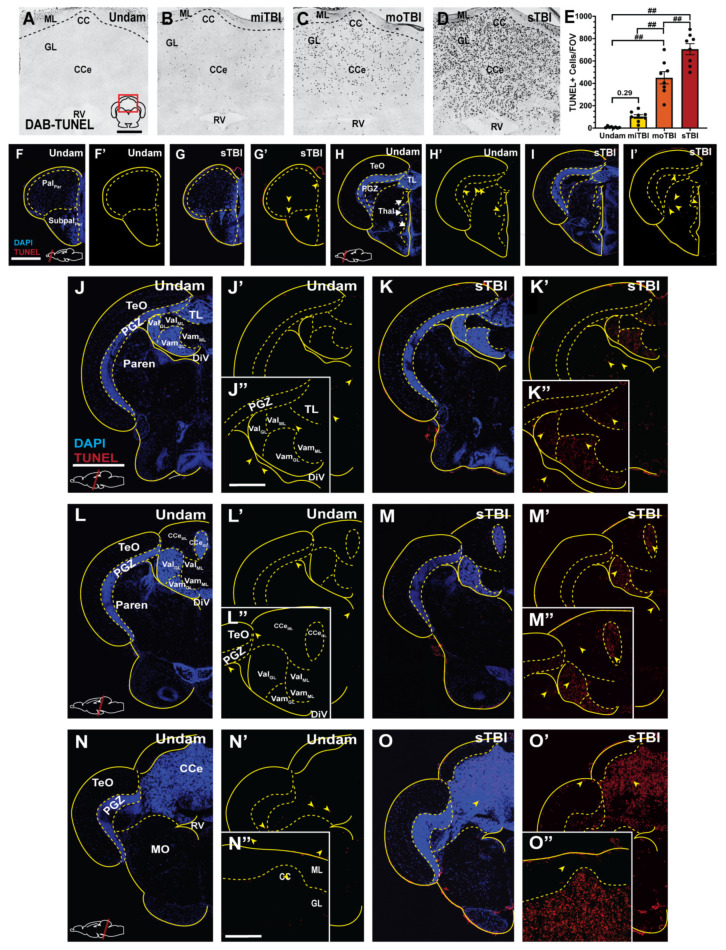
Cell death is severity-dependent and radiates out from the impact zone as a gradient. (**A**–**D**) Brightfield images of coronal cerebellar (CCe) sections stained with DAB-TUNEL 16 hpi. Prominent DAB TUNEL-positive cells were observed in the CCe in a severity-dependent manner. (**E**) Quantification of the number of DAB-TUNEL-positive cells/section revealed significant increases in the number of TUNEL-positive cerebellar cells following moTBI and sTBI (n = 8). (**F**–**O”**) Confocal images of coronal sections, from rostral to caudal across the neuroaxis, stained with fluorescent TUNEL and DAPI-labeled at 16 hpi (**J**–**O**). Apoptotic cell death, emanating from the impact zone, was observed widely across the neuroaxis. The red box in panel A shows the relative position of the image on the appropriate brain cross-section. Solid lines denote tissue boundaries, while dotted lines denote internal anatomic boundaries. Cerebellar crest, CC, corpus cerebelli, CCe, diencephalic ventricle, DiV, granule cell layer, GL, molecular layer, ML, medulla oblongata, MO, parenchyma, Paren, periventricular grey zone, PGZ, optic tectum, TeO, torus longitudinalis, TL, rhombencephalic ventricle, RV, medial valvula cerebelli, Vam, lateral valvula cerebelli, Val. Scale bars: (**A**) = 100 µm, for panels (**A**–**D**,**F**) = 200 µm, for panels (**F**–**I’**,**J**) = 500 µm, for panels (**J**–**O’**,**J”**) = 200 µm, for panels (**J”**–**M”**,**N”**) = 100 µm, for panels (**N”**,**O”**). Mean ± SEM is depicted in (**E**). Statistical analyses were performed with a One-way ANOVA followed by a Tukey’s post-hoc test. ## *p* < 0.01.

We quantified the number of TUNEL-positive dying cells in brains following each of the three injury levels. In the cerebellum, the region primarily located within the impact zone, there was not a significant difference in the number of TUNEL-positive cells following miTBI (miTBI:103.62 ± 16.77, *p* = 0.29, n = 8, Figure 4B,E) relative to undamaged controls (7.25 ± 2.57 cells, n = 8, Figure 4A,E). This minor damage/cell death following miTBI is similar to human miTBI patients displaying negative CT/MRI scans [59,60]. However, there were significant increases in TUNEL-positive cells in moTBI (450 ± 54.65 cells, *p* < 0.01, n = 8, Figure 4C,E) and sTBI (705.5 ± 49.54 cells, *p* < 0.01, n = 8, Figure 4D,E) relative to undamaged controls. Additionally, there were significantly greater numbers of TUNEL-positive cells between miTBI and moTBI (*p* < 0.01) and between moTBI and sTBI (*p* < 0.01), which is consistent with the scalable nature of the damage model.

**Figure 5:**
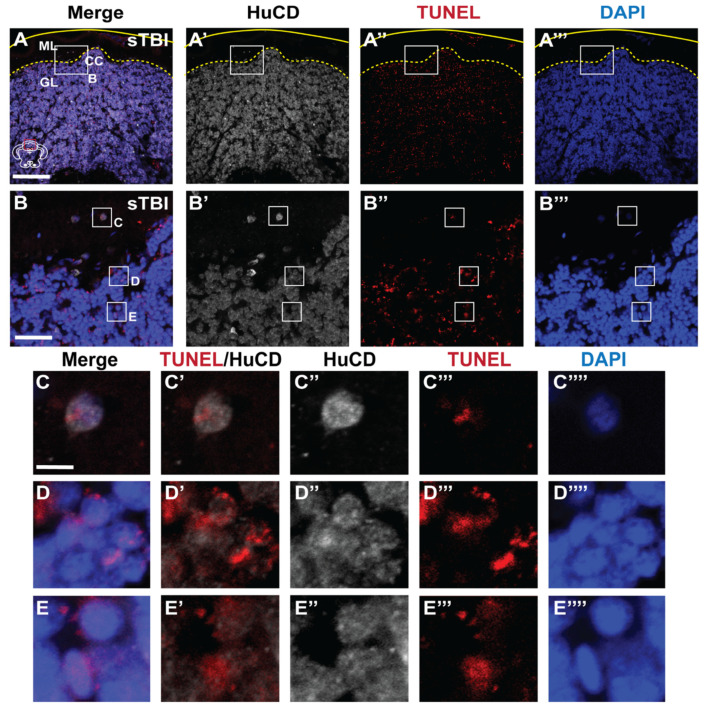
Apoptotic cell-death in cerebellar neurons. (**A**–**B’’’**) Confocal images of coronal cerebellar sections stained with pan-neuronal marker HuCD (gray), fluorescent TUNEL (red), and DAPI (DAPI) at 16 h follow a sTBI. Apoptotic cell death was observed in the epicenter of the impact zone, in the densely packed granule layer of the cerebellum. (**C**–**E’’’’**) High magnification confocal images revealed colabelling of HuCD, TUNEL, and DAPI. The white box in panels (**A**–**A’’’**) represent the region that is shown in corresponding panels (**B**–**B’’’**). Lettered white boxes in panels (**B**–**B’’’**) denote subsequent panels at higher magnification. A solid line denotes tissue boarder, dotted line denotes boarder between molecular and granule layer of the CCe. Cerebellar crest, CC, granule cell layer, GL, molecular layer, ML. Scale bars, (**A**) = 100 µm, for panels (**A**–**A’’’**,**B**) = 50 µm, for panels (**B**–**B’’’**,**C**) = 15 µm, for panels (**C**–**E’’’’**).

We hypothesized a blunt-force trauma would result in a diffuse injury and cell death, unlike the focal injury associated with a penetrating TBI models. Therefore, we examined the entire neuroaxis of sTBI fish for apoptotic cell death following blunt-force TBI. We observed only a few TUNEL-positive cells in the most rostral parts of the brain (Figure 4F,F’,G,G’), though TUNEL-positive cells became more evident in the rostral parts of the midbrain approximately 0.5–1 mm outside of the impact zone (Figure 4I,I’). However, the most prominent labeling was observed in the granule cell layers of the medial and lateral valvula cerebelli (Vam and Val, Figure 4K,K’,M–M”), and the corpus cerebelli (CCe, Figure 4M–M”,O–O”), regions with high densities of cell bodies. Importantly, the number of TUNEL-positive cells increased in a gradient emanating from the epicenter of the impact zone. At a lower occurrence, we observed TUNEL-positive labeling in the Periventricular Gray Zone (PGZ, Figure 4K–K”,M–M’’,O,O’) and other regions with high density of neuronal cell bodies located laterally away from the impact zone. We further examined apoptotic cell death in the cerebellum, the epicenter of the impact zone, and co-stained with the pan-neuronal marker HuCD. We observed double-positive TUNEL/HuCD labeling across the cerebellum of sTB fish (Figure 5A–A’’’), to include large TUNEL/HuCD double positive cells within the Purkinje layer (Figure 5B–C’’’’) and smaller double-labeled cells within the granule layer (Figure 5B,D–E’’’’). These data suggest that blunt-force trauma results in a diffuse injury accompanied by apoptotic neuronal cell death that occurs in a gradient radiating outward from the impact zone.

### 3.4. TBI Results in Sensorimotor Impairments and Associative Learning and Memory Deficits with Rapid Recovery

**Figure 6:**
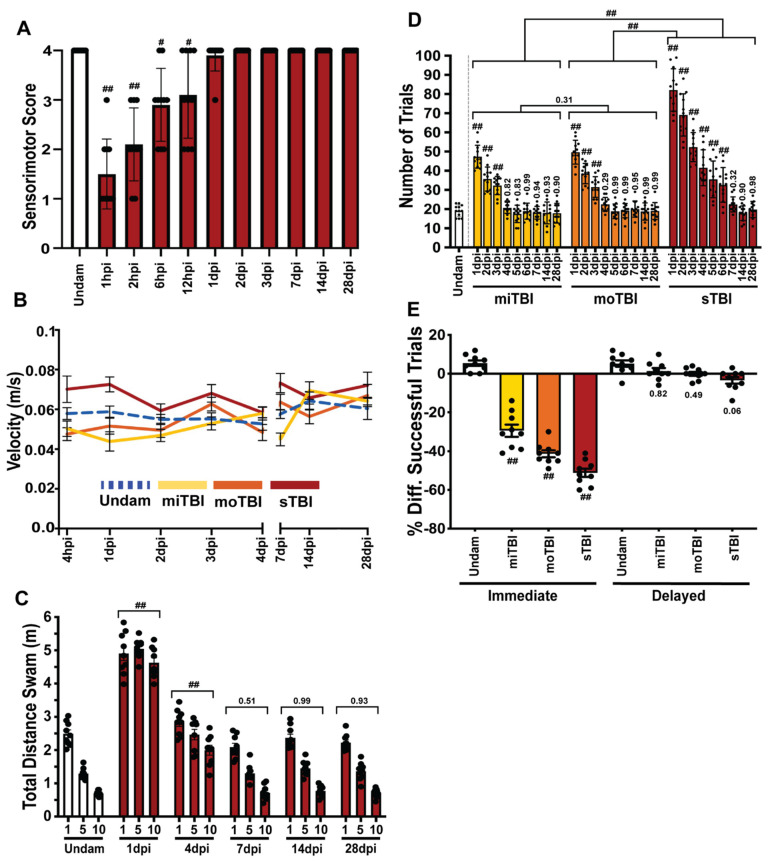
Injury-induced sensorimotor and cognitive deficits are severity-dependent with rapid recovery. (**A**) Sensorimotor coordination (plotted as a sum of four independent sensorimotor features involving swimming orientation and adverse tactile stimulus, Table 1) was significantly impaired following sTBI for up to 12 hpi compared to the pre-injury response (n = 10). (**B**) The unprovoked swim velocity over time was not significantly different between undamaged controls and all three severity models from 4 hpi to 28 dpi (n = 15). (**C**) Quantification of the swim distance of undamaged and sTBI fish following the startle response across iterations 1, 5, and 10 at 1, 4, and 7 dpi. TBI-damaged fish displayed cognitive deficits in this non-associative learning assay (n = 9). (**D**) Quantification of associative learning, using the shuttle box assay, of undamaged fish and all TBI severities from 1–28 dpi measuring the number of trials to master the assay (n = 12). All three damage severities resulted in a significant reduction in learning that returned to normal between 4–7 dpi. (**E**) Quantification of immediate- and delayed-recall of associative learning of undamaged and all three TBI severities using the shuttle box assay. All three TBI categories (miTBI, moTBI, sTBI) resulted in both learning and recall deficits (n = 9). Mean ± SEM is depicted in (**A**–**C**,**E**), while standard deviation is shown in (**D**). Statistical analyses were performed with either Two-way ANOVA, or a One-way ANOVA followed by a Tukey’s or Dunnett’s multiple comparison post-hoc test. # *p* < 0.05, ## *p* < 0.01.

A blunt-force injury often results in sensorimotor and cognitive impairment, with deficits increasing with injury-severity in human TBI populations [15,61,62]. Following TBI, we evaluated swim orientation, analogous to gait, and response to an adverse tactile stimulus to collectively assess sensorimotor coordination (Supplementary Table S1). Prior to injury, fish swam horizontally and parallel to the tank bottom/water surface, never breached the surface, and consistently displayed nocifensive, escape, and avoidance behaviors in response to an adverse tactile stimulus. At 1–2 hpi following sTBI, fish displayed disoriented swim profiles that were noticeably inclined or tilted and often breached the surface of the water with the most rostral portion of their head (Table 1). These abnormal swimming behaviors were absent by 6 hpi. Responses to tactile stimuli were impaired for longer durations following sTBI. All injured fish displayed and continued to display nocifensive behaviors at all timepoints, while at 1 hpi few fish displayed escape behaviors, and none avoided the stimulus (Table 1). Escape behaviors remained significantly impaired through 2 hpi (*p* = 0.05, Table 1) and returned to normal by 1 dpi, while avoidance behaviors remained significantly impaired through 12 hpi (*p* = 0.05, Table 1) and returned to normal by 2 dpi. Each behavior was individually scored and then collectively summed as a total sensorimotor score. Relative to preinjury, sTBI fish displayed significantly impaired sensorimotor scores at 1 hpi (*p* < 0.01) through 12 hpi (*p* < 0.05) and returned to near pre-damaged response by 1 dpi (*p* = 0.99, Figure 6A). Thus, following sTBI, our model induces orientation and sensorimotor coordination deficits similarly seen in human TBI patients.

We next evaluated habituation, a primitive non-associative learning response defined by a gradually decreased response over time to a continuous or repetitive stimulus [63]. In our model, the impact zone was centered over the intersection of the midbrain and hindbrain. The startle response is a well characterized behavioral assay that is predominantly initiated and executed by reticulospinal neurons in the hindbrain and also the granule neurons of the cerebellum, which have been shown to contribute to classical fear response [64]. Because the non-associative startle response quantifies total swim distance and velocities following the startle [41], we first assessed the general locomotion and swim profiles of undamaged and TBI fish for potential locomotor dysfunction. There was no significant difference in the swim velocity between undamaged and either miTBI, moTBI, or sTBI fish from 4 hpi to 28 days post-injury (dpi) (Figure 6B, n = 15). This suggests that locomotor function is not significantly affected in our TBI model.

To measure the startle response, a 100 g weight was dropped from water level next to the tank every 60 s for 10 iterations. During iteration 1, undamaged control fish responded with rapid bursts of swimming, with an initial velocity of 0.26 m/s ± 0.02 m/s and a total distance travelled of 2.48 m ± 0.11 m before returning back to relatively sedentary state within 22 s ± 1.59 s (Figure 6C, Table 2). However, undamaged fish quickly displayed signs of habituation, as their initial bursts of swimming shortened to 15 s ± 1.24 s and 10 s ± 0.6 s for iterations 5 and 10, respectively. Similarly, initial velocities of 0.19 m/s ± 0.01 m/s and 0.14 m/s ± 0.01 m/s and average swimming distances of 1.29 m ± 0.03 m and 0.69 m ± 0.03 m for were reduced for iterations 5 and 10, respectively (Figure 6C, Table 2).

Following the startle, sTBI fish at 1 dpi responded with an initial velocity of 0.25 m/s ± 0.01 m/s during iteration 1, which was similar to undamaged fish (Table 2). However, sTBI fish at 1 dpi did not display any signs of habituation as the initial velocities for iterations 5 and 10 were 0.23 m/s ± 0.02 m/s, and 0.25 m/s ± 0.01 m/s, respectively, and exhibited persistent bursts of increased swimming activity that lasted longer than 30 s during all iterations (Table 2). Consequently, sTBI fish at 1 dpi swam a significantly greater distance than undamaged control fish for iterations 1, 5, and 10, averaging 4.90 m ± 0.26 m, 5.04 m ± 0.16 m, and 4.62 m ± 0.18 m, respectively (Figure 6C). The sTBI fish at 4 dpi swam a total distance that gradually decreased from 2.84 m ± 0.15 m during iteration 1 to 2.46 m ± 0.2 m and 1.99 m ± 0.19 m during iterations 5 and 10, respectively (Figure 6C), although there was no statistically significant difference between iterations 1, 5, and 10. While the sTBI fish began to habituate at 4 dpi, unlike at 1 dpi, they still displayed increased swimming velocities for 21–24 s (Table 2). In contrast, sTBI fish at 7 dpi displayed habituation of the startle response similar to undamaged controls in every metric, including decreased swim velocity, decreased swim distance, and returning to a sedentary state from iterations 1 to 10 (Figure 6C, Table 2). Startle responses resembling undamaged behaviors persisted through 14 and 28 dpi (Figure 6C, Table 2). These data demonstrate that following blunt-force injury, sTBI fish display an impairment in habituation, a non-associative form of learning, that rapidly recovers to near undamaged control levels by 7 dpi and persists through 28 dpi.

Because the sTBI resulted in cell death across the brain, we asked if this would result in broader cognitive dysfunction. Therefore, we tested associative learning and memory, higher-level cognitive tasks that are modulated in teleosts in part by the medial- and lateral-dorsal pallium of the telencephalon [65,66], a region adjacent to our impact zone. To assess associative learning, we used a modified shuttle box assay [39], with a visual stimulus and an electrical shock as an adverse stimulus prompting learning by negative reinforcement [40]. Because the shuttle box assay relies on fish recognizing a visual stimulus, we first assessed survival of retinal neurons in sTBI fish. The number of TUNEL-positive cells in each retinal layer were the same in both sTBI and undamaged fish (Supplementary Figure S1A). In addition, we did not detect a significant number of proliferating Müller glia in either undamaged or sTBI fish (Supplementary Figure S1B), which is a typical response following retinal damage [23].

Undamaged control fish required an average of 19 ± 1.09 trials (Figure 6D) to master the assay (completing 5 consecutive positive trials without the negative reinforcement). Following injury, miTBI (47 ± 1.88 trials, *p* < 0.01, n = 12, Figure 6D), moTBI (51 ± 3.93 trials, *p* < 0.01), and sTBI fish (82 ± 2.98 trials, *p* < 0.01) all required significantly more trials to master the shuttle box assay relative to undamaged control fish at 1 dpi. At 2 dpi and 3 dpi, significant learning deficits persisted for all three TBI severities (Figure 6D). By 4 dpi, the average number of trials for miTBI and moTBI to master the shuttle box assay decreased to a level similar to undamaged fish (Figure 6D). In contrast, sTBI fish continued to display significant, but declining, deficits through 6 dpi, and returned to the undamaged control levels by 7 dpi, where they remained through 28 dpi (Figure 6D). Thus, this learning deficit correlated with the level of damage severity, both in magnitude (number of trials to master the assay) and duration before returning to control levels.

While humans with miTBI display little physiological pathologies, difficulties with short-term memory are a common feature [67]. We assessed whether our model recapitulated both immediate- and delayed recall of a learned behavior in undamaged and TBI treated fish using the shuttle box assay [40]. For immediate recall, the fish learned a behavior and then 4 h later were either undamaged or subjected to a TBI and then retested for the behavior learned 4 h before the TBI. Undamaged fish displayed immediate recall, with an increase of 5.44% ± 2.13% in successful trials when retested 4 h following testing period 1 (Figure 3E). In contrast, we observed a significant decrease in successful trials post-injury, suggesting TBI fish exhibited difficulty in immediate recall. Specifically, miTBI fish exhibited a −29.44% ± 2.17% decrease in immediate recall (*p* < 0.01), and the deficits were further decreased to −41.33% ± 1.37% (*p* < 0.01) and −51.16% ± 1.66% (*p* < 0.01) following moTBI and sTBI, respectively (Figure 6E, n = 9). For delayed recall, the fish learned a behavior and then 4 days later were either undamaged or subjected to a TBI and then retested for the behavior learned 4 days before the TBI. The miTBI and moTBI fish demonstrated delayed recall comparable to undamaged fish (Figure 6E), with sTBI fish displaying a slight, near significant decrease (*p* = 0.06). Similar to human TBI patients [15,68], zebrafish experience significant deficits in immediate recall following TBI with deficits increasing in a severity-dependent manner, while delayed recall retention was relatively unaffected.

### 3.5. TBI Induces Cell Proliferation across the Neuroaxis in a Severity-Dependent Manner

**Figure 7:**
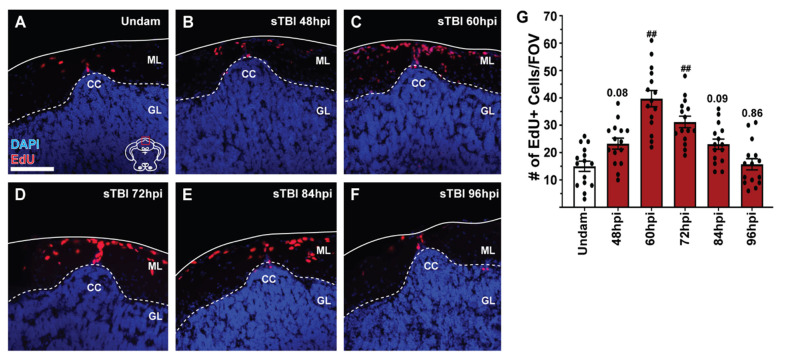
Blunt-force TBI induces cell proliferation at the cerebellar crest. (**A**–**F**) Confocal images of coronal cerebellar sections of undamaged and sTBI fish that were IP-injected with EdU 12 h prior to collection. EdU-labelled cells (red) are present radiating from the cerebellar crest in all panels. (**G**) Quantification of the number of EdU-positive cells at the CC at various timepoints following sTBI (n = 15). Increased EdU labeling, relative to undamaged fish, was observed as early as 48 hpi, with peak proliferation at 60 hpi. Solid lines in (**A**–**F**) denote tissue boundary, while dotted lines denote internal anatomical boundaries. Cerebellar crest, CC, granule cell layer, GL, molecular layer, ML. Scale bar = 100 µm for (**A**–**F**). Mean ± SEM is depicted in (**G**). Statistical analysis was performed using a One-way ANOVA followed by a Tukey’s post-hoc test. # *p* < 0.05, ## *p* < 0.01.

Previously, zebrafish were shown to have a robust injury-induced proliferative response that is localized to the injury site [22,25,27]. However, the sizable gradient of apoptotic cell death that radiated from the epicenter of the impact zone (Figure 4), coupled with the recovery of learning deficits over several days (Figure 6C,D), led us to examine the extent of cell proliferation across the neuroaxis in a severity-dependent manner. The timing of peak cell proliferation was assessed by intraperitoneally (IP) injecting undamaged and sTBI fish with EdU at 12 h intervals from 36 to 84 hpi (Figure 7A–F) and examining EdU incorporation in brains at 12 h post injection (48, 60, 72, 84, and 96 hpi). Compared to undamaged controls, we observed the largest significant increase in the number of EdU-positive cells in sTBI brains at 60 and 72 hpi (Figure 7C,D,G) in the molecular layer (ML) adjacent to the cerebellar crest (CC), the epicenter of the impact zone.

**Figure 8:**
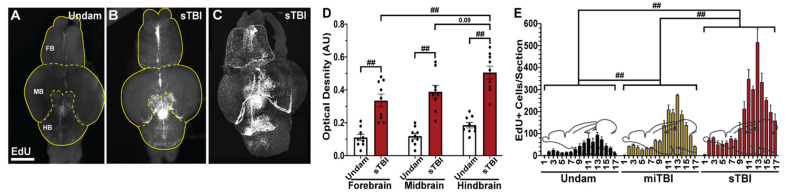
Blunt-force TBI results in increased proliferation in the brain. (**A**,**B**) Dorsal view of isolated and chemically-cleared undamaged (**A**) and sTBI (**B**) brains labeled with EdU and analyzed by fluorescent microscopy. The position of the forebrain (FB), midbrain (MB), and hindbrain (HB) are delineated. (**C**) Chemically-cleared sTBI brain imaged by lightsheet microscopy. (**D**) Quantification of optical density of EdU fluorescence by bulk brain region revealed significant increases in fluorescence in all sTBI regions relative to undamaged brains, with the largest increase seen in the hindbrain near the impact epicenter (n = 9). (**E**) Quantification of the total number of EdU-positive cells per section across the neuroaxis of undamaged, miTBI, and sTBI fish (n = 4). Significant increases in proliferation were observed in miTBI and sTBI brains relative to undamaged brains from the rostral tip of the olfactory bulb to caudal tip of the cerebellum. Scale bar = 500 µm. Mean ± SEM is depicted in D and E. Statistical analyses were performed with either a One-way ANOVA or Two-way ANOVA followed by a Tukey post-hoc test. ## *p* < 0.01.

To initially obtain a broad overview of the extent of proliferation along the neuroaxis following TBI, we examined whole brains from undamaged and sTBI fish that were EdU injected at 48 hpi and collected 12 h later at the peak of cell proliferation (Figure 7G). Cleared brains were stained and EdU incorporation was assessed using an epifluorescent microscope and confirmed by lightsheet fluorescence microscopy. EdU incorporation in undamaged fish revealed constitutive pockets of basal levels of cell proliferation across the brain (Figure 8A). However, following injury, large increases of EdU incorporation were observed within the impact zone that expanded across the brain (Figure 8B,C). To compare the injury-induced proliferation across the neuroaxis, the optical density was determined for the forebrain, midbrain, and hindbrain of undamaged and sTBI fish. Relative to undamaged fish, we observed significant increases in optical density in sTBI fish across the forebrain (Undam: 0.11 ± 0.013, sTBI: 0.33 ± 0.038 A.U., *p* < 0.01, n = 9, Figure 8A,B,D), midbrain (Undam: 0.12 ± 0.017, sTBI: 0.39 ± 0.039 A.U., *p* < 0.01, n = 9, Figure 8A,B,D), and hindbrain (Undam: 0.19 AU ± 0.017, sTBI: 0.52 AU ± 0.017, *p* < 0.01, n = 9, Figure 8A,B,D).

To further investigate the distribution of proliferating cells within the different brain regions, EdU-injected brains (n = 4) of undamaged controls, miTBI, and sTBI fish were serially sectioned and the number of EdU-positive cells in each section was quantified (Figure 8E). EdU-positive cells were found throughout the entire undamaged brain, with the forebrain and rostral sections of the midbrain containing low numbers of EdU-positive cells, while larger numbers of EdU-positive cells were present in the caudal midbrain and hindbrain. In both miTBI and sTBI fish, proliferation was significantly increased across the entire neuroaxis compared to undamaged brains (*p* < 0.01, Figure 8E). Proliferation was also significantly further elevated across the entire sTBI brain relative to miTBI (*p* < 0.01). Thus, even following a miTBI, which exhibits minimal pathophysiological deficits, the damage induces a significant cell proliferation response.

**Figure 9:**
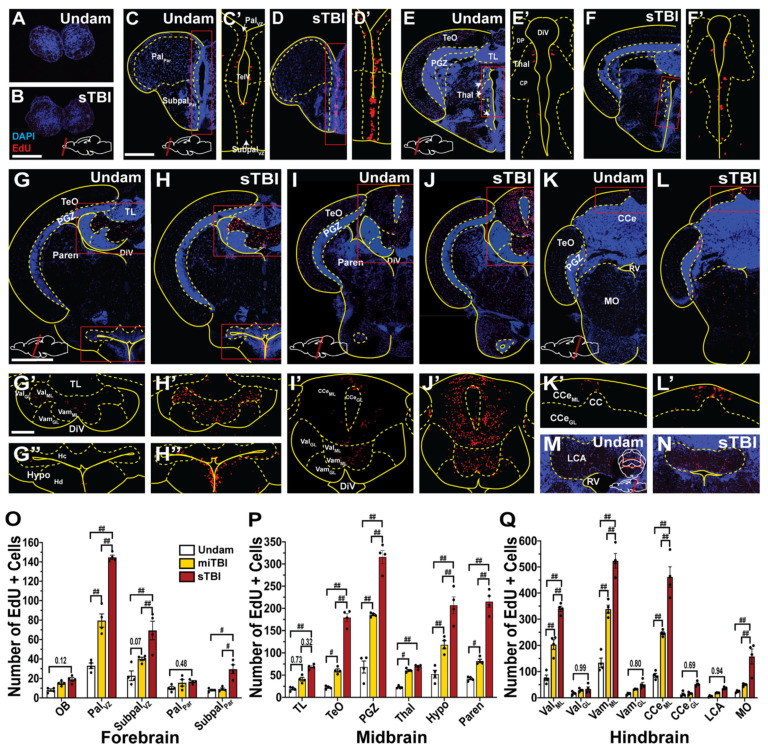
Injury-induced proliferation across the neuroaxis is region-specific and severity-dependent. (**A**–**N**) Coronal brain sections of undamaged and sTBI fish from the rostral aspect of the olfactory bulb to the caudal aspect of the lobus caudalis cerebelli. Red boxed regions in (**C**–**L**) are shown across the midline at a higher magnification in the corresponding prime and double prime panels. (**O**–**Q**) Quantification of the number of EdU-positive cells in brain subregions in undamaged, miTBI, and sTBI fish (n = 4). Solid lines in (**A**–**N**) denote tissue boundary, while dotted lines denote internal anatomical boundaries. Central posterior thalamic region, CP, corpus cerebelli, CCe, granule cell layer of corpus cerebelli, CCe_GL_, molecular layer of corpus cerebelli, CCe_ML_, diencephalic ventricle, DiV, dorsal posterior thalamic region, DP, lobus caudalis cerebelli, LCA, medulla oblongata, MO, olfactory bulbs, OB, parenchyma of pallium, Pal_Par_, ventricular/subventricular zone of pallium, Pal_VZ_, parenchyma of midbrain, Paren, periventricular grey zone of tectum optic, PGZ, rhombencephalic ventricle, RV, telencephalic ventricle, TeV, optic tectum, TeO, thalamus, Thal, torus longitudinalis, TL, parenchyma of subpallium, Subpal_Par_, ventricular/subventricular zone of subpallium, Subpal_VZ_, granule cell layer of lateral valvula cerebelli, Val_GL_, molecular layer of lateral valvula cerebelli, Val_ML_, granule cell layer of medial valvula cerebelli, Vam_GL_, molecular layer of medial valvula cerebelli, Vam_ML_. Scale bars: (**B**) = 100 µm, for panels (**A**–**C**) = 200 µm, for panels (**C**–**F**’,**G**) = 500 µm, for panels (**G**–**L**,**G’**) = 200 µm, for panels (**G’**–**L’**,**M**,**N**). Mean ± SEM is depicted in (**O**–**Q**). Statistical analyses were performed with a Two-way ANOVA followed by a Sidik’s multiple comparison test. # *p* < 0.05, ## *p* < 0.01.

A more detailed analysis of each section revealed specific subregions with increased EdU incorporation in TBI fish relative to undamaged controls. At the most rostral aspect of the brain, and furthest from the impact zone, the olfactory bulb displayed increased proliferation (Undam: 8.5 ± 2.05, miTBI: 16 ± 1.45, sTBI: 19 ± 2.89 cells). However, these increases in the olfactory bulb were not significant relative to undamaged fish (sTBI *p <* 0.12, n = 4, Figure 9A,B,O), suggesting that the blunt-force injury did not reach the olfactory bulbs. More caudally, significantly greater numbers of EdU-positive cells were present in miTBI fish along the ventricular/subventricular zone (VZ) of the pallium (Pal_vz_: miTBI: 79.5 ± 6.81 cells, *p* < 0.01, n = 4, Figure 9O) compared to undamaged controls (Pal_vz_: Undam: 33 ± 2.85 cells). The number of EdU-positive cells along the pallium VZ was further elevated in sTBI brains (Pal_vz_: sTBI: 144.75 ± 2.25 cells, *p* < 0.01, n = 4, Figure 9C,D) relative to undamaged controls and also between the miTBI and sTBI fish (*p* < 0.01, Figure 9O). Additionally, the VZ of the subpallium displayed significantly greater EdU incorporation in both miTBI (Subpal_vz_: 40 ± 1.77 cells, *p* < 0.01, n = 4) and sTBI (Subpal_vz_: 69.25 ± 9.56 cells, *p* < 0.01, n = 4) relative to undamaged controls (Subpal_vz_: Undam: 22.75 ± 4.97 cells, Figure 9C–D’,O, Supplementary Figure S2), and between the miTBI and sTBI fish (*p* < 0.01, Figure 9O). In contrast, the telencephalon parenchyma possessed only a few EdU-positive cells in controls, with the miTBI and sTBI not showing a significant increase in EdU labeling, with the exception of the subpallium of sTBI fish relative to undamaged controls (Subpal_Par_: sTBI: 29.5 ± 4.55 cells, *p* < 0.05, n = 4, Figure 9C–D’,O, Supplementary Figure S2).

Further caudally, approaching the impact zone, the midbrain was divided into six neuroanatomical regions, as defined by the neuroanatomical atlas of the adult zebrafish brain [69], including the Thalamus (Thal), Hypothalamus (Hypo), and the Periventricular grey zone (PGZ). Following miTBI and sTBI, there was a significantly greater number of EdU-positive cells within several regions relative to undamaged controls and across injury-severities. The Thal exhibited significantly more EdU-labeled cells in miTBI (60.5 ± 2.03, *p* < 0.05, n = 4) and sTBI (70 ± 2.28 cells, *p* < 0.01, n = 4, Figure 9E–F’,P) fish than in undamaged controls (22.75 ± 1.43 cells). Similarly, Hypo possessed significantly greater numbers of EdU-positive cells in miTBI fish (118 ± 9.1 cells, *p* < 0.01, n = 4) and sTBI fish (206.75 ± 18.89, *p* < 0.01, Figure 9G,G”,H,H”,P) than undamaged controls (52.25 ± 8.62 cells). Furthermore, in the midbrain, we observed the largest number EdU-labelled cells following TBI in the PGZ, which spans nearly the entirety of the midbrain. Relative to the undamaged PGZ (68.25 ± 13.59 cells), there were significantly greater numbers of EdU-labeled cells in the miTBI (185.5 ± 2.21 cells, *p* < 0.01, n = 4) and sTBI PGZ (315.75 ± 14.98, *p* < 0.01, n = 4, Figure 9G,H,P).

The hindbrain, which was the epicenter of the impact zone, was divided into the molecular and granular layers of the Lateral valvula cerebelli (Val_ML_, Val_GL_), Medial valvula cerebelli (Vam_ML_, Vam_GL_), Corpus cerebelli (CCe_ML_, CCe_GL_), Lobus caudalis cerebelli (LCA), and the Medulla oblongata (MO). The hindbrain possessed the largest basal levels of EdU-positive cells per neuroanatomical region in undamaged brains and the largest number of EdU-labeled cells following injury (Figure 8, Supplementary Figure S2). Following both miTBI and sTBI, significantly more EdU-positive cells were present in the molecular layers compared to undamaged controls and across severities (Figure 9G–L,Q, Supplementary Figure S2). Areas with prominent increases in EdU-positive cells following injury included the Val (miTBI: 203.25 ± 18.28, *p* < 0.01, n = 4, sTBI: 339.75 ± 9.97 cells, *p* < 0.01, n = 4) and the Vam (miTBI:338.5 ± 15.21, *p* < 0.01, sTBI:523.75 ± 27.91, *p* < 0.01, n = 4) relative to undamaged brains (Val_ML_: 76 ± 11, Vam_ML_: 134 ± 17.7 cells Figure 9G–J’,Q). One of the regions with the largest number of EdU-positive cells following injury was the cerebellar crest (CC) of the CCe. The undamaged CC displayed 85.5 ± 7.57 EdU-positive cells, while miTBI exhibited a significantly greater number (246.5 ± 5.73 cells, *p* < 0.01, n = 4), with even greater numbers in sTBI CC (461.5 ± 39.08 cells, *p* < 0.01, n = 4, Figure 9K–L’,Q, Supplementary Figure S2). The most caudal and ventral sections of miTIB and sTBI brains displayed significantly greater numbers of EdU-labeled cells in the MO (miTBI: 49.5 ± 5.26 cells, *p* < 0.01, sTBI: 157.75 ± 28.54 cells, *p* < 0.01, n = 4) relative to undamaged controls (24 ± 3.91 cells, Figure 9K,L,Q). However, we observed no significant increases in EdU labeling in the LCA following either miTBI or sTBI relative to undamaged controls (Figure 9M,N,Q). Collectively, these data reveal that following injury, constitutive neurogenic regions significantly upregulated cell proliferation in a severity-dependent manner and the cell proliferation radiated beyond the impact zone.

### 3.6. TBI Results in Injury-Induced Cerebellar Proliferation, Progenitor Migration, and Differentiation

The Upper Rhombic Lip and the Cerebellar Recessuss, which collectively corresponds to the area (CC) in the adult hindbrain, has been heavily studied in development as a proliferative region that produces neuronal progenitors that migrate and differentiate into most cerebellar cell types, including the most common, granule cell neurons [70]. As the fish age, the CC continues to generate basal levels of progenitor cells that migrate into the granule cell layer of the cerebellum and differentiate into granule cell neurons [71]. Because the CC exhibits significant increases in proliferation upon blunt-force trauma (Figure 7A–F and Figure 9K–L’), we sought to identify the migration and proliferative source of cerebellar progenitors, as well as the fate of these cells.

**Figure 10:**
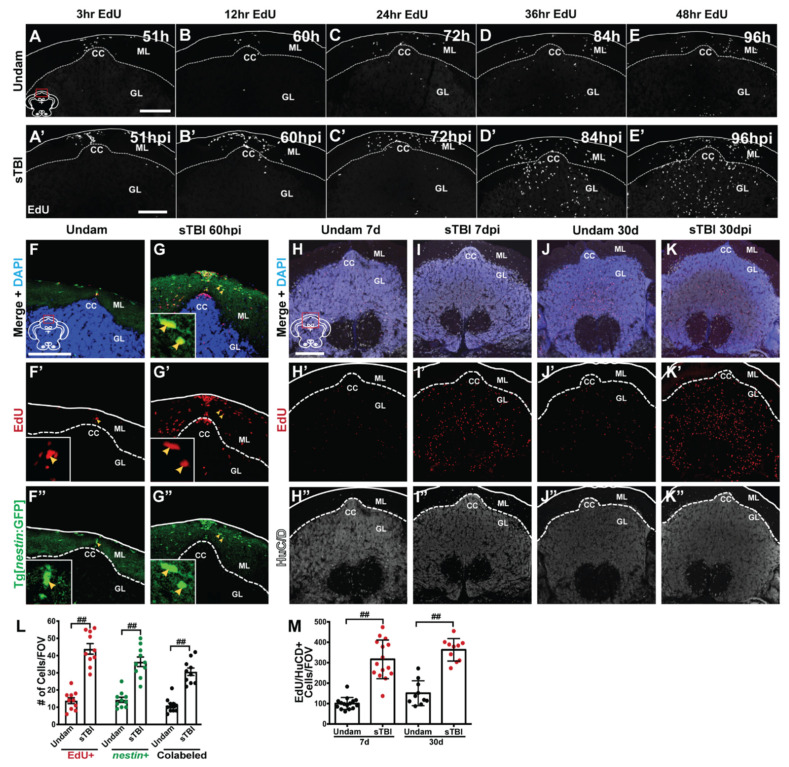
Blunt-force TBI induces cell proliferation, migration, and differentiation in the cerebellum. (**A**–**E’**) Coronal cerebellar sections of sTBI fish (**A’**–**E’**) that were IP-injected with EdU at 48 hpi and collected 51, 60, 72, 84, and 96 hpi to identify the migration pattern of injury-induced proliferative cells. Control undamaged fish (**A**–**E**) were also injected and brains assessed at similar intervals as sTBI fish. Coronal cerebellar sections of undamaged (**F**) and sTBI (**G**) Tg[*nestin*:GFP] fish with high magnification insets (**F’–F’’**,**G’–G”**) that were IP-injected with EdU 12 h prior to collection at 60 hpi with colabeling of EdU and Tg[*nestin*:GFP] (yellow arrowheads). Coronal cerebellar sections of undamaged (**H**–**H’’**,**J**–**J’’**) and sTBI fish (**I**–**I’’**,**K**–**K’’**) that were IP-injected with EdU at 48 and 60 hpi to capture early onset and peak proliferative events and collected at either 7 (**H**–**I’’**) or 30 dpi (**J**–**K’’**) and costained with HuCD. (**L**) Quantification of the number of EdU-positive, *nestin*:GFP-positive, or colabeled cells for experiments in representative images (**F**–**G”**) (n = 10). (**M**) Quantification of the number of EdU/HuCD colabeled cells for experiments in representative images (**H**–**K”**) (n = 15). Solid lines in (**A**–**K”**) denote tissue boundary, while dotted lines denote internal anatomical boundaries. Cerebellar crest, CC, granule cell layer, GL, molecular layer, ML. All scale bars = 100 µm. Mean ± SEM is depicted in L and M. Statistical analyses were performed with either a One-way ANOVA or Two-way ANOVA followed by a Tukey’s post-hoc test. ## *p* < 0.01.

To assess cell migration, undamaged and sTBI fish were IP-injected with EdU at 48 hpi and collected at 51, 60, 72, 84, and 96 hpi. Following injury, the CC displayed increased EdU incorporation relative to undamaged controls (Figure 10A–E’). These EdU-positive cells appeared to originate at the apical aspect of the CC (Figure 10A’,B’) and then migrated apically and laterally through the molecular layer at 60–72 hpi (Figure 10B’,C’). EdU-positive cells then moved ventrally into the granule cell layer starting at 84 hpi and heavily infiltrated the granule cell layer by 96 hpi (Figure 10D’,E’).

During cerebellar development, the progenitors arose from populations of *nestin:EGFP* and *ptf1a:DsRed*-expressing cells [70,72]. However, following partial lateral excision of the adult zebrafish cerebellum, a significant increase of *nestin:EGFP*/PCNA double-positive cells was observed, while only limited numbers of *ptf1a:DsRed*/PCNA-positive cells were present [27]. We assessed EdU incorporation in the Tg[*nestin*:GFP] line, during peak proliferation following blunt-force TBI. Undamaged and sTBI Tg[*nestin*:GFP] fish were IP-injected with EdU at 48 hpi and collected 12 h later. Undamaged fish displayed low levels of EdU-incorporation (14.8 ± 1.72 cells, n = 10) and *nestin*:GFP-positive cells (14.6 ± 1.51 cells, n = 10), with most cells expressing both markers (11 ± 1.35 cells, n = 10, Figure 10F–F”,L). However, following injury, we observed a significant increase in the number of EdU-positive cells (43.9 ± 3.03 cells, *p* < 0.01, n = 10) and *nestin*:GFP-positive cells (36.4 ± 2.77 cells, *p* < 0.01, n = 10) at the CC relatively to the undamaged control. Furthermore, there was a significant increase in the number of colabeled cells (30.6 ± 2.18 cells, Figure 10G–G”,L, *p* < 0.01, n = 10) at 60 hpi compared to undamaged controls.

We next asked if these EdU-positive cells that infiltrated the granule cell layer differentiated into neurons. Undamaged and sTBI fish were given EdU pulses at 48 and 60 hpi and we quantified the colocalization of EdU and the pan-neuronal marker HuCD at short (7 dpi) and long (30 dpi) recovery timepoints. Undamaged fish displayed basal levels of EdU/HuCD-colabeled cells in the granule cell layer of the cerebellum at 7 dpi (100.13 ± 7.6 cells, Figure 10H–H”,M) and the number of colabeled cells remained statistically unchanged at 30 d (151 ± 18.8 cells, *p* = 0.24, n ≥ 10, Figure 10J–J”,M). Following sTBI, we observed a significant increase in the number of EdU/HuCD-colabeled cells within the cerebellar granule cell layer relative to undamaged fish at both 7 dpi (316.36 ± 24.49 cells, *p* < 0.01, n = 15, Figure 10I–I”,M) and 30 dpi (356 ± 18.03, *p* < 0.01, n = 10, Figure 10K,K”,M). Similar to undamaged fish, we did not see a statistical difference in the number of EdU/HuCD-colabeled cells in sTBI fish between 7 dpi and 30 dpi (*p* = 0.46), suggesting that following injury, cells proliferate, migrate into the granule cell layer of the cerebellum, stably regenerate differentiated neurons, and then repress further regeneration.

### 3.7. Sonic Hedgehog Regulates Injury-Induced Proliferation in the Cerebellum

**Figure 11:**
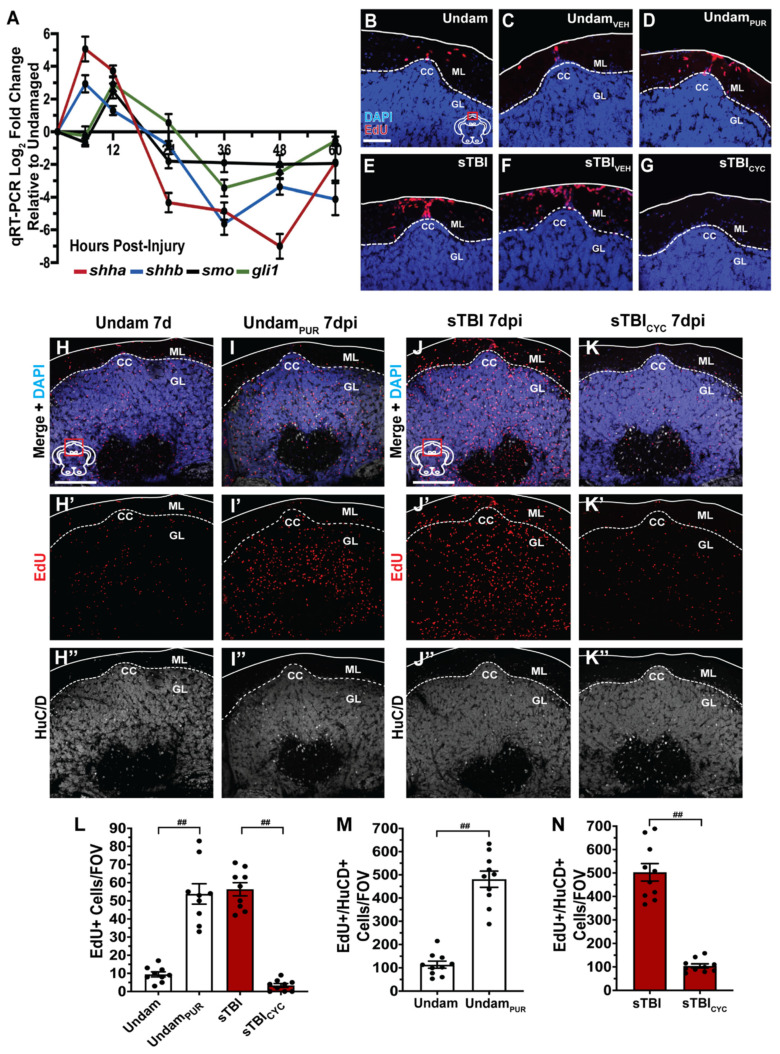
Shh regulates cerebellar proliferation and differentiation following injury. (**A**) Expression of Shh pathway genes by qRT-PCR reveals that the *shha* and *shhb* mRNAs are upregulated by 4 hpi in the top 1/3rd of the cerebellum, while *gli* and *smo* expression increased by 12 hpi (5 pooled cerebellums/group, n = 3 groups). (**B**–**G**) Coronal cerebellar sections of the CC of undamaged (**B**–**D**) and sTBI fish (**E**–**G**) that were IP-injected with EdU and either vehicle (**C**,**F**), the Smo agonist purmorphamine (**D**), or the Smo antagonist cyclopamine (**G**). (**H**,**H”**–**I**,**I”**) Coronal cerebellar sections of undamaged fish that were either untreated (**H**–**H’’**) or purmorphamine-treated (**I**–**I’’**) that were IP-injected with EdU and collected at 7 dpi. Coronal cerebellar sections of sTBI fish that were either untreated (**J**–**J’’**) or cyclopamine-treated (**K**–**K’’**) that were IP-injected with EdU and collected at 7 dpi. (**L**) Quantification of the number of EdU-positive cells at the CC in undamaged and sTBI fish with Shh modulation (n = 9). (**M**,**N**) Quantification of the number of EdU/HuCD double-positive cells in the granule cell layer of the cerebellum in undamaged and sTBI fish with Shh modulation (n = 10). Solid lines in (**B**–**K”**) denote tissue boundary, while dotted lines denote internal anatomical boundaries. Cerebellar crest, CC, granule cell layer, GL, molecular layer, ML. All scale bars = 100 µm. Mean ± SEM is depicted in (**L**–**N**). Statistical analyses were performed with either a One-way ANOVA or Two-way ANOVA followed by a Tukey’s post-hoc test. ## *p* < 0.01.

Sonic hedgehog (Shh) is a well characterized mitogen involved in development and regeneration and was demonstrated to play a critical role in regulating progenitors and neuronal regeneration following CNS trauma [73,74]. Therefore, we hypothesized that TBI-induced proliferation at the CC was regulated by Shh signaling and examined the temporal expression of Shh pathway components. We performed qRT-PCR to assess *shha*, *shhb*, *smo*, and *gli1* expression using RNA collected from the most dorsal third of undamaged and sTBI isolated cerebellums across early timepoints following blunt force trauma to the peak of cell proliferation (6, 12, 24, 36, 48, 60 hpi). Relative to undamaged control cerebellums, both *shha* and *shhb* RNAs (Shh ligands) were highly upregulated by 6 hpi, followed by increased expression of *smo* (Shh receptor) and *gli1* (downstream effector) by 12 hpi (Figure 11A).

To evaluate the influence of Shh signaling to stimulate TBI-induced cerebellar proliferation, undamaged controls were administered either 10 μM purmorphamine, a Smoothened (Smo) agonist, or vehicle control every 12 h for 48 h, then coinjected with EdU at 48 h, and collected at 60 h. Low basal levels of EdU-positive cells were observed at the CC in both untreated (9.75 ± 1.16 cells, Figure 11B) and vehicle-treated (Figure 11C) undamaged fish. In contrast, purmorphamine-treated undamaged fish exhibited a significant increase in the number of EdU-labeled cells at the CC (54.12 ± 5.51 cells, *p* < 0.01, n = 9, Figure 11D, L), relative to untreated controls. Interestingly, purmorphamine-induced proliferation in undamaged fish was similar to the proliferative response observed in untreated sTBI fish (*p* = 0.95, Figure 11L). Conversely, sTBI fish were administered either vehicle control or 2 mM cyclopamine, a Smo antagonist, at 4 hpi to account for early pathway activation and at 12, 24, 36, and 48 hpi, followed by coinjection with EdU at 48 hpi and collected 12 h later. Robust EdU-incorporation was observed in both untreated sTBI (56.5 ± 4.07 cells, Figure 11E) and vehicle-treated sTBI fish (Figure 11F). However, cyclopamine-treated sTBI fish had significantly fewer EdU-positive cells at the CC (4.25 ± 0.77 cells, *p* = 0.01, n = 9, Figure 11G,L) relative to the untreated control sTBI fish. Importantly, there was no significant difference in the number of EdU-positive cells between untreated undamaged controls and cyclopamine-treated sTBI fish (*p* = 0.60, Figure 11L), suggesting that Shh plays a role in TBI-induced cerebellar proliferation.

To further investigate the role of Shh in neuronal regeneration following a blunt-force TBI, we examined the differentiation and production of new granule cell neurons in the cerebellum. Undamaged fish were IP-injected with purmorphamine every 12 h for 48 h, IP-injected with EdU at 48 and 60 h, and collected at 7 d to examine EdU and HuCD colabeling in the CC granule cell layer. Untreated, undamaged fish exhibited low numbers of differentiated HuCD neurons (113.3 ± 15.27 cells, n = 10, Figure 11H–H”,M), while purmorphamine-treated undamaged fish displayed a significantly greater number of EdU/HuCD double-positive cells in the CC granule cell layer (492 ± 32.52 cells, *p* < 0.01, n = 10, Figure 11I–I”,M). We also examined sTBI fish that were either uninjected or injected with cyclopamine at 4, 12, 24, 36, and 48 hpi. Both groups of sTBI fish were also IP-injected with EdU at 48 hpi and 60 hpi, collected at 7 dpi, and the number of EdU and HuCD double-positive cells were quantified in the CC granule cell layer. Following injury, the uninjected sTBI fish possessed a large number of EdU/HuCD double-positive cells in the granule cell layer (508.2 ± 37.7 cells, n = 10, Figure 11J–J”,N). However, cyclopamine-treated sTBI fish exhibited significantly fewer double-positive cells (104.2 ± 8.7 cells, *p* < 0.01, n = 10, Figure 11K–K”,N) relative to the uninjected sTBI fish. Collectively, these data demonstrated that Shh signaling plays a role in regulating TBI-induced proliferation and the generation of HuCD-labeled neurons in the CC granule cell layer.

## 4. Discussion

Traumatic brain injuries produce a breadth of both acute and chronic pathologies [75], which largely correlate with injury severity. The current study describes a rapid and simple TBI model that utilizes the most common mechanism to induce human TBI: blunt-force trauma [1,76]. We extensively characterized a scalable blunt-force injury model to examine mild, moderate, or severe TBIs in zebrafish, including the heterogeneity of severity-dependent injury-induced pathologies and the potential mechanisms underlying innate neuronal regeneration in the zebrafish brain. While the Marmarou weight drop is simple and rapid, it lacks the precision found in controlled cortical impact or lateral fluid percussive models, which are extensively studied in rodents [77]. Nevertheless, our results indicate that the zebrafish model induces many TBI sequelae analogous to those reported in the human population, including BBB disruption, neuroinflammatory response, and cognitive issues [3,48,49,78]. Consistency between human and zebrafish TBI-induced pathology makes zebrafish a useful tool to not only study the pathology and subsequent recovery post-TBI, but also provides the unique opportunity to study neuronal regeneration, which could reveal novel therapies for human TBI patients.

Previously, a blunt-force TBI model was described in adult zebrafish [32]. Our procedure was similar, except to dissipate the energy of the blunt-force trauma and to prevent cranial fractures in sTBI fish, we placed a small steel disc on the skull of the fish before dropping the weight, as is often done with rodents. The energy applied by Maheras et al. [32] was reported to be 35 mJ, which was over three-orders of magnitude greater than our calculated energies 1.33 mJ (miTBI), 2.08 mJ (moTBI), and 2.94 mJ (sTBI). The difference is that Maheras et al. [32] calculated their energy based on a fixed velocity of the falling ball, rather than an accelerating velocity, where the ball starts from a stationary position. If we used their parameters and calculated an accelerating velocity for the falling ball, then their energy is 0.35 mJ, which is 26% less than our mild blunt-force TBI (their steel ball had a mass of 0.33 g relative to our ball’s mass of 1.5 g). Furthermore, the 35 mJ reported by Maheras et al. [32] is approaching the 40 mJ calculated from dropping a 20 g weight from a height of 20 cm onto a rat skull [79], which would likely crush the zebrafish skull. Thus, we feel that our miTBI is similar to the reported mild TBI energy reported by Maheras et al. [32].

Maheras et al. [32] did not report any significant phenotypic responses, outside of a learning and memory impairment using a T-box shoaling assay, or regenerative recovery in their mild blunt-force TBI. However, our study expanded the characterization to include several features of human TBI-induced pathologies across three levels of severity (mild, moderate, and severe) in adult zebrafish. Because classification of human TBI severity is often diagnosed with a collective, rather than a singular metric [3,48,49,78], we employed a variety of analogous tests to examine the breadth of zebrafish TBI pathologies. These diagnostic pathologies included injury-induced seizures and death, edema, BBB disruption, neuroinflammation, sensorimotor deficits, neuronal cell death, and cognitive impairments. While we did not exhaustively examine all known pathologies ascribed to human TBIs, our model validated a wide array of phenotypes that increased in severity with increasing levels of blunt force trauma. In humans, miTBI is the most reported injury [76], with many individuals experiencing little to no effects [59]. Similarly, our model demonstrated that following a miTBI, many injury-induced phenotypes (seizures, recovery rate, edema, vascular injury, and neuronal cell death) were not significantly different relative to undamaged controls, while there was a significant decrease in cognitive ability relative to undamaged controls.

Many of the injury-induced pathologies that we identified within 1 hpi could be early signs of the gradient of cell death we detected beginning at 16 h following sTBI, which emanated from the impact zone to more rostral portions of the brain. However, neuronal damage and possibly necrotic cell death likely occurred before 16 hpi and this could be associated with the early injury-induced pathologies we observed. While cell death was limited in the telencephalon, which is the analogous to the hippocampus and the location of many behaviors and cognitive ability [65,66], this region likely experienced severe disruption due to the significant cognitive deficits. While we did not measure learning within 16 hpi, we did examine the immediate recall of the fish at 4 hpi. In this case, we found that all three levels of TBI resulted in immediate memory deficits, suggesting that their cognitive function was negatively affected and supporting the idea that the telencephalon experienced sufficient damage. Alternatively, damage to the cerebellum, which has been implicated in fear learning and the escape response [64], may negatively affect a cerebellar brain circuit and the cognitive deficits. Additionally, while the neuronal cell death we observed is likely not the cause of the pathological and cognitive deficits, it is a significant outcome of the blunt-force trauma.

One of the major reasons to study TBI in zebrafish is its innate neuronal regenerative capacity across a wide range of tissues, which cannot be studied in mammalian TBI models [80,81]. While neuronal regeneration in zebrafish has been examined previously, most studies of injury-induced proliferation have focused on focal injuries and the surrounding injury site [24,25,27]. One of the few studies describing a proliferative response outside of the immediate injury site, Amamoto et al. [82] reported BrdU-positive cells in the rostral portion of the adult axolotl telencephalon after surgically removing a portion of the dorsal pallium. Similarly, Lindsey et al. [44] described increased proliferation beyond the stab wound site in the adult zebrafish telencephalon. However, they quantified the proliferative response as a measure of optical density and combined multiple regions of the brain into large bulk areas: the forebrain, midbrain, and hindbrain limiting the analysis of proliferation in subregions. Our bulk proliferative findings (Figure 8A–D) are largely in agreement with Lindsey et al. [44]. Additionally, we provide a comprehensive and quantitative comparative analysis of the proliferation across the neuroaxis from the rostral tip of the olfactory bulb to the caudal aspect of the rhombencephalon, including multiple subregions, following mild and severe blunt force TBI. This significant cell proliferation response, even following a miTBI, suggests that the blunt force trauma either induces widespread neuronal damage outside of the impact zone or generates a broad damage signal to initiate cell proliferation.

We focused on the cerebellum for the impact zone and our regeneration response because the zebrafish cerebellum is well characterized developmentally, with committed neuronal progenitors originating in the Upper rhombic lip and symmetrically dividing to produce the neuronal diversity in the cerebellum [83,84]. This developmental program persists into adulthood and mediates active neurogenesis throughout the zebrafish adult life [19,71]. Furthermore, the large quiescent neurogenic niche in the cerebellum (Figure 8) [71,85] has previously been studied in the context of localized injury-induced proliferation. Partial surgical excision of the cerebellum in adult zebrafish resulted in increased proliferation at the cerebellar crest and the proliferating cells subsequently migrated in a water fountain fashion to repopulate the cerebellar granule cell layer [19,27,71]. We similarly identified the cerebellar crest as a source of proliferating cells that migrate to the cerebellar granule cell layer and differentiate into neurons. The identity of this proliferation source, the various mechanisms utilized to induce and regulate this regeneration response, the spectrum of neuronal types that can be regenerated, and the recovery of functional circuits are obvious questions to be explored further.

The Shh pathway is essential for early development and neurogenesis [86] Recently, Shh was shown to be critical for zebrafish neuronal regeneration following trauma in other parts of the CNS [73,74]. Similarly, we demonstrated that blunt-force trauma to the cerebellum induced the expression of *shh* pathway genes. However, the bulk RNA-Seq dataset reported by Maheras et al. [32] did not reveal an upregulation of genes associated with Shh signaling. This was likely due to Maheras et al. [32] performing their bulk RNA-Seq on RNA isolated from brains at 3 and 21 dpi. The increased expression of Shh signaling genes that we observed using qRT-PCR reached peak expression by 12 hpi. By 60 hpi, which is our latest timepoint and 12 h prior to the bulk RNA-Seq dataset, the expression of *shha*, *shhb*, and *smo* had all decreased below their baseline expression level and *gli1* had returned to its undamaged expression level. We also confirmed that Shh signaling is necessary for the subsequent proliferation response at the cerebellar crest as demonstrated by cyclopamine treatment eliminating nearly all injury-induced proliferation at the cerebellar crest and decreased numbers of EdU/HuCD double-positive cells 7dpi. Furthermore, Shh activation in undamaged fish, by purmorphamine exposure, provoked a proliferative response at the cerebellar crest similar to the amount of proliferation observed following blunt-force trauma, which differentiated into HuC/D-positive neurons in the cerebellar granule cell layer. It remains to be determined what role, if any, Shh signaling has on the other TBI-induced pathologies in zebrafish.

## 5. Conclusions

1. The modified TBI model for zebrafish is scalable for mild, moderate, and severe injury.
2. Zebrafish blunt-force TBI produces heterogeneous phenotypes replicating human injury.
3. Injury results in cognitive deficits that rapidly recover within 7 days.
4. Following injury, significant proliferation is observed across the entire brain.
5. Shh regulates injury-induced proliferation in the cerebellum.

## Figures and Tables

**Table 1:** Sensorimotor coordination is briefly impaired following blunt-force TBI. Quantification of swim orientation (Tilt) and behavioral responses to three adverse tactile stimulus responses (Pain Stimuli, Escape, and Avoidance) as defined in the zebrafish behavior catalog (Kalueff, et al., 2013) for 10 fish pre-injury and followed through several time points to 28 dpi (n = 10).

Time (n = 10)	Tilt Avg ZBC 1.83, 1.164, 1.175	Pain Stimuli Avg ZBC 1.104	Escape Avg ZBC 1.5, 1.52	Avoidance Avg ZBC 1.12	Total Score
Undam	1	1	1	1	4
sTBI 1 hpi	0.4	1	0.1	0	1.5
sTBI 2 hpi	0.7	1	0.4	0	2.1
sTBI 6 hpi	1	1	0.7	0.2	2.9
sTBI 12 hpi	1	1	0.7	0.4	3.1
sTBI 1 dpi	1	1	1	0.9	3.9
sTBI 2 dpi	1	1	1	1	4
sTBI 3 dpi	1	1	1	1	4
sTBI 7 dpi	1	1	1	1	4
sTBI 14 dpi	1	1	1	1	4
sTBI 28 dpi	1	1	1	1	4

**Table 2:** Blunt-force TBI induces non-associative learning impairments that rapidly recover. Quantification of initial swim velocities and recovery time following startle onset of undamaged and sTBI fish that were evaluated for startle response at iterations 1, 5, and 10 of the startle stimulus across multiple dpi (n = 9).

Group n = 9	Iteration		
	1	5	10
Undam Startle Velocity	0.256 ± 0.02 m/s	0.190 ± 0.01 m/s	0.138 ± 0.01 m/s
(Recovery Time)	(22 ± 1.59 s)	(15 ± 1.24 s)	(10 ± 0.60 s)
sTBI 1 dpi Startle Velocity	0.253 ±0.01 m/s	0.233 ± 0.02 m/s	0.246 ± 0.01 m/s
(Recovery Time)	(30 s+)	(30 s+)	(30 s+)
sTBI 4 dpi Startle Velocity	0.246 ± 0.01 m/s	0.231 ± 0.01 m/s	0.216 ± 0.01 m/s
(Recovery Time)	(23 ± 1.02 s)	(24 ± 1.88 s)	(21 ± 0.95 s)
sTBI 7 dpi Startle Velocity	0.236 ± 0.02 m/s	0.153 ± 0.01 m/s	0.100 ± 0.01 m/s
(Recovery Time)	(21 ± 1.25 s)	(16 ± 1.14 s)	(13 ± 1.03 s)
sTBI 14 dpi Startle Velocity	0.248 ± 0.02 m/s	0.171 ± 0.01 m/s	0.144 ± 0.02 m/s
(Recovery Time)	(23 ± 1.12 s)	(17 ± 1.09 s)	(12 ± 1.31 s)
sTBI 28 dpi Startle Velocity	0.251 ± 0.01 m/s	0.182 ± 0.01 m/s	0.124 ± 0.01 m/s
(Recovery Time)	(21 ± 1.71 s)	(15 ± 1.22 s)	(11 ± 0.89 s)

## Data Availability

Data are contained within this article and the Supplementary Materials.

## Supplementary Materials

The following are available online at https://www.mdpi.com/article/10.3390/biomedicines9080861/s1, Figure S1: Blunt-force TBI does not induce retinal damage, Figure S2: sTBI induces increased cell proliferation across the neuroaxis, Table S1: Sensorimotor ethogram.
