# Supplementary material for: Zebrafish Blunt-Force TBI Induces Heterogenous Injury Pathologies That Mimic Human TBI and Responds with Sonic Hedgehog-Dependent Cell Proliferation across the Neuroaxis
Source: Biomedicines. 2021 Jul 22;9(8):861. doi: 10.3390/biomedicines9080861 (PMC8389629; doi:10.3390/biomedicines9080861)
Supplement: Supplementary file 1 [file biomedicines-09-00861-s001.zip › biomedicines-1282735-supplementary.pdf]

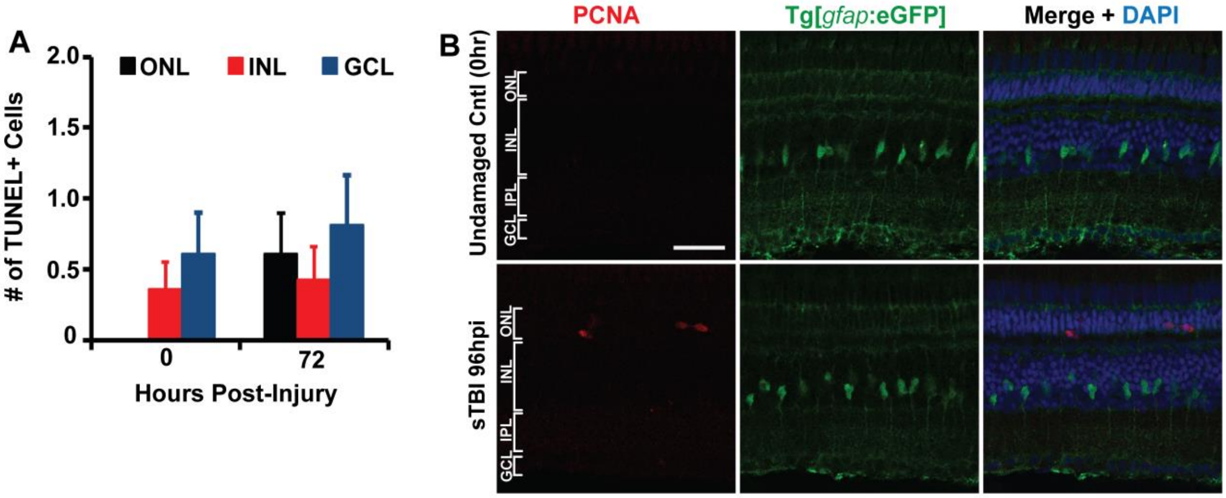

Supplemental Figure S1. Blunt-force TBI does not induce retinal damage. (A) Quantification of the total number of TUNEL-positive cells in the retinal ONL, INL, and GCL at 0 hr

(undamaged) and 72 hpi of sTBI fish eyes. There was no significant increase in the number of TUNEL-positive cells following blunt-force TBI relative to undamaged retinas. (B) Confocal images of retinal sections from Tg[*gfap*:eGFP] undamaged and sTBI fish to assess damage-induce Müller glia proliferation. There were equivalent numbers of PCNA-positive cells in the undamaged controls and the sTBI fish. Ganglion cell layer, GCL, inner nuclear layer, INL, inner plexiform layer, IPL, outer nuclear layer, ONL. Scale bar=25 µm. Mean ±SEM is depicted in A. Statistical analysis was performed with a Student's *t*-test comparing the different nuclear layers between undamaged control and sTBI.

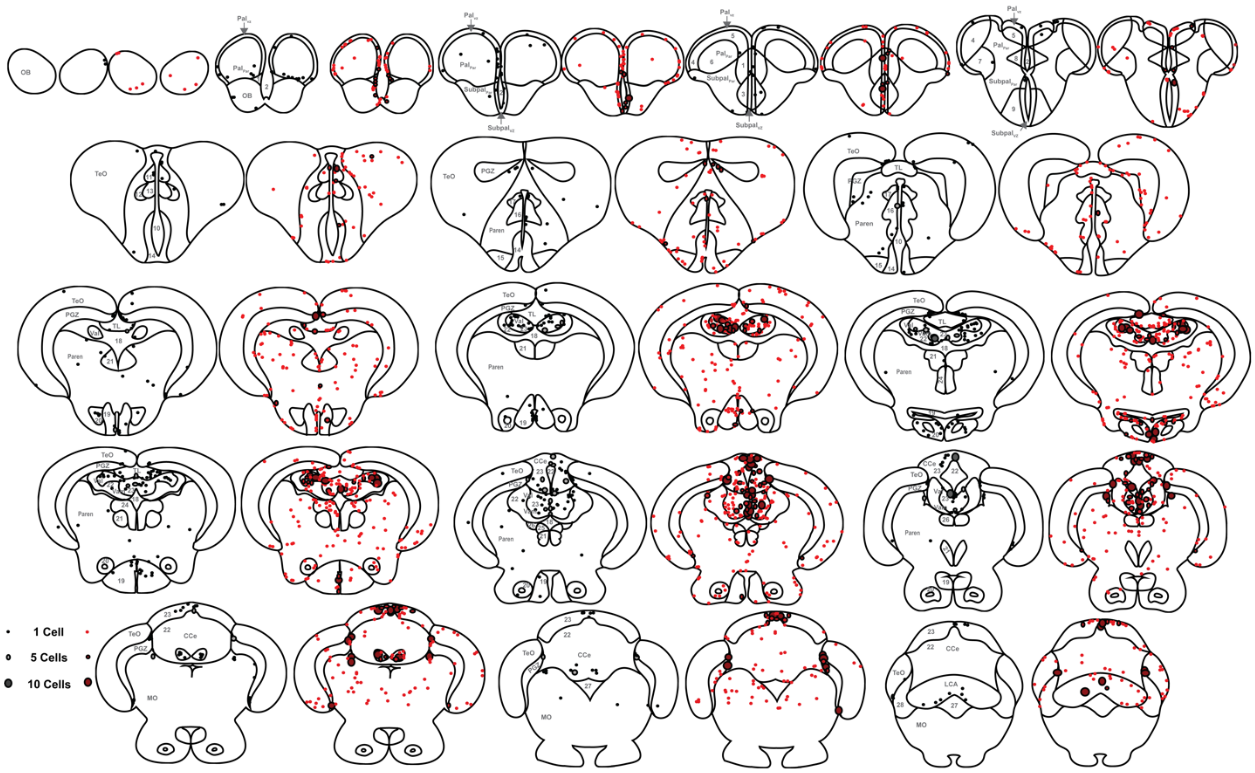

Supplemental Figure S2. sTBI induces increased cell proliferation across the neuroaxis. (A) Representative diagrams of serial coronal brain sections from a single undamaged brain (black label) and sTBI brain (red label) injected with EdU and collected at the peak of cell proliferation at 60 hpi. The size of the red or black dot represents the number of EdU-labelled cells (1, 5, and 10) in that approximate location. Major Regions: CCe, corpus cerebelli, Hypo, hypothalamus, LCA, lobus caudalis cerebelli, MO, medulla oblongata, OB, olfactory bulb, Pal_{Par}, parenchyma of pallium, Pal_{VZ}, ventricular/subventricular zone of pallium, Paren, parenchyma, PGZ, periventricular grey zone, $Subpal_{Par}$, parenchyma of subpallium, $Subpal_{VZ}$, ventricular/subventricular zone of subpallium, Teo, tectum opticum, Thal, thalamus, TL, torus longitudinalis, Val, lateral division of valvula cerebelli, Vam, medial division of valvula cerebelli. Minor Regions: 1, dorsal nucleus of subpallium, 2, telencephalic ventricle, 3, ventral nucleus of subpallium, 4, lateral zone of pallium, 5, medial zone of pallium, 6, central zone of pallium, 7, posterior zone of pallium, 8, supracommissural nucleus of subpallium, 9, anterior parvocellular preoptic nucleus, 10, diencephalic ventricle, 11, anterior thalamic nucleus, 12, ventrolateral thalamic nucleus, 13, ventromedial thalamic nucleus, 14, ventral zone of periventricular hypothalamus, 15 lateral zone of periventricular hypothalamus, 16, central posterior thalamic nucleus, 17, dorsal posterior thalamic nucleus, 18, tectal ventricle, 19, caudal zone of periventricular hypothalamus, 20, dorsal zone of periventricular hypothalamus, 21, medial longitudinal fascicle, 22, granule layer, 23, molecular layer, 24, oculomotor nucleus, 25, trochlear nucleus, 26, griseum centrale, 27, rhombencephalic ventricle, 28, eminentia granularis.

Pain Stimuli<br>**ZBC 1.104**	Escape<br>**ZBC 1.5, 1.52**	Avoidance<br>**ZBC 1.12**	Tilt<br>**ZBC 1.83, 1.164, 1.175**
1= Tail-beating	1= Changes direction from stimuli	1= Swim to opposite side of tank	1= Horizontal swimming parallel to water surface and bottom of tank
0= No tail-beating	0= No change in direction from stimuli	0= Removes from stimuli but does not swim to opposite side of tank	0= Angular, inclined, or surfaced swimming

Supplemental Table S1. Sensorimotor Ethogram. Prior to, and following injury at multiple repeated time points, fish were scored binary on swim orientation and nocifensive, escape, and avoidance behavior following an adverse tactile stimulus.
